# The molecular connection of histopathological heterogeneity in hepatocellular carcinoma: A role of Wnt and Hedgehog signaling pathways

**DOI:** 10.1371/journal.pone.0208194

**Published:** 2018-12-04

**Authors:** Anindita Tripathy, Sudhir Thakurela, Manoj Kumar Sahu, Kanishka Uthanasingh, Manas Behera, Amrendra Kumar Ajay, Ratna Kumari

**Affiliations:** 1 Disease Biology Lab, KIIT School of Biotechnology, KIIT University, Bhubaneswar, India; 2 Broad Institute of MIT and Harvard, Cambridge, MA, United States of America; 3 Department of Stem Cell and Regenerative Biology, Harvard University, Cambridge, MA, United States of America; 4 Department of Gastroenterology & Hepatobiliary Sciences, IMS & SUM Hospital, Bhubaneswar, India; 5 Brigham and Women’s Hospital, Harvard Medical School, Boston, MA, United States of America; University of Hong Kong, HONG KONG

## Abstract

**Background:**

Hepatocellular carcinoma (HCC) is leading cause of cancer-related mortality and is categorized among the most common malignancies around the world. It is a heterogeneous tumor, which shows significant degree of histopathological heterogeneity. Despite the apparent histopathological diversity there has been very little distinct correlation between histopathological features and molecular aberrations particularly when it comes to the expression level of Wnt and Hh pathway molecules. The role of Wnt and Hh pathways in relation to HCC behavior viz. histopathological heterogeneity and aggressiveness is not known. Determining the sequential molecular changes and associated histopathological characteristic during HCC initiation, promotion, and progression would probably lead to a better treatment and prognosis.

**Methods:**

N-Nitrosodiethylamine (DEN) induced HCC model in male Wistar rats were established to study the expression level of Wnt and Hh pathway molecules during different stages of hepatocarcinogenesis. Their expression levels were checked at mRNA and protein levels at initiation, promotion, and progression stages of HCC. The expression levels of Wnt and Hh pathway molecules were correlated with biospecimens of HCC patients of different stages.

**Results:**

In the present study we identified the comprehensive change in the expression pattern of Wnt and Hh pathway molecules in DEN induced rodent hepatocarcinogenesis model. Our results demonstrate that β-catenin /CTNNB1 plays important role in tumor initiation and promotion by stimulating tumor cell proliferation. The activated Wnt signaling in early stage of HCC is associated with well-differentiated histological pattern. The Hh activity although activated during the initiation stage but is significantly increased during the early promotion stage of hepatocarcinogenesis. The increased activity of both Wnt & Hh pathways during promotion stage is associated with moderately-differentiated histological pattern and was simultaneously linked with an increased expression of MMP9. Furthermore, our data demonstrated that during the progression stage Wnt pathway is modestly down-regulated but the Hh pathway activity sustained which in turn is associated with aggressive and invasive phenotype and poorly-differentiated histopathology.

**Conclusion:**

Our data uncovers the grade related expression of Wnt and Hh pathway molecules and the potential utility of these molecular signatures in daily clinical practice is to decide best therapy according to patients characteristic. Additionally, our data offer insight into the interaction between Wnt and Hh pathways which triggers HCC development and progression.

## Background

Hepatocellular carcinoma (HCC) is leading cause of cancer-related mortality and is categorized among the most common malignancies around the world[1]. HCC is a morphologically and clinically heterogeneous tumor and it also shows a significant degree of histopathological heterogeneity[2–4]. This heterogeneous nature of HCC pose serious problem for its therapeutic management. Particularly, there is obvious and frequent histopathological changes observed at different stages of HCC. The varied histopathological changes are associated with the change in invasion and metastatic properties of HCC and can also alter the response of HCC towards any therapy. The invasion and metastatic properties of cells in HCC varies from early stage to late stage. Indeed the stage of HCC along with its differentiation status had been a central aspect for clinical evaluation of HCC behavior. The range of cellular differentiation in HCC extends from well-differentiated to poorly-differentiated types and contains varied morphological subtypes. Depending upon tumor differentiation grade along with other factors like cancer stage, patients age, and underlying disease condition a treatment regimen is selected. Studies have shown that tumor differentiation grade is significantly correlated to the stage of tumors as per TNM (Tumor-Node-Metastasis) system in colorectal cancer[5]. Similarly, other investigators have also shown the correlation between stage and grade of tumors[6–7].

Despite the apparent histopathological diversity there has been very little distinct correlation between histopathological features and molecular aberrations particularly when it comes to the expression level of Wnt and Hh pathway molecules. Determining the sequential molecular changes and associated histopathological characteristic during HCC initiation, promotion, and progression would probably lead to a better treatment and prognosis.

One of the important diagnostic features for almost all malignancies including HCC is cytological properties which includes the cellularity, arrangement-pattern, and nucleo-cytoplasmic details. Based on these properties HCC can be categorized into well-differentiated, moderately-differentiated, poorly-differentiated, and undifferentiated lesions[8]. The widespread molecular nature of HCC across different grades is not known. Two major signaling pathways found to be deregulated in HCC are Wnt and Hedgehog (Hh) pathways. Wnt and Hh signaling pathways play key roles in embryogenesis, tissue patterning, morphogenesis, stem-cell maintenance, and tumorigenesis[9–10]. The involvement of these pathways in the regeneration of liver after injury is well documented[11–12]. The Wnt pathway is known for its contribution to HCC[13–14]. Similarly the Hh pathway is found to be frequently activated in HCC[15–16]. However the role of these pathways in relation to HCC behavior viz. histopathological heterogeneity and aggressiveness is not known.

In the present study we identified the comprehensive change in the expression pattern of Wnt and Hh pathway molecules in DEN induced rodent hepatocarcinogenesis model. The differential expression of Wnt and Hh pathway molecules could discriminate the differences in the histopathological grades from pre-neoplastic lesion to advanced HCC. Simple microscopic examination is not enough to differentiate between different grades of HCC. A significant difference in the molecular signature of each histopathological grade during hepatocarinogenesis would open a window for grade specific treatment option. Additionally, molecular signatures associated with specific grades would in turn improve the histopathological grading system.

## Results

### DEN induced HCC is associated with expanding nodular pattern during hepatocarcinogenesis

**Fig 1:**
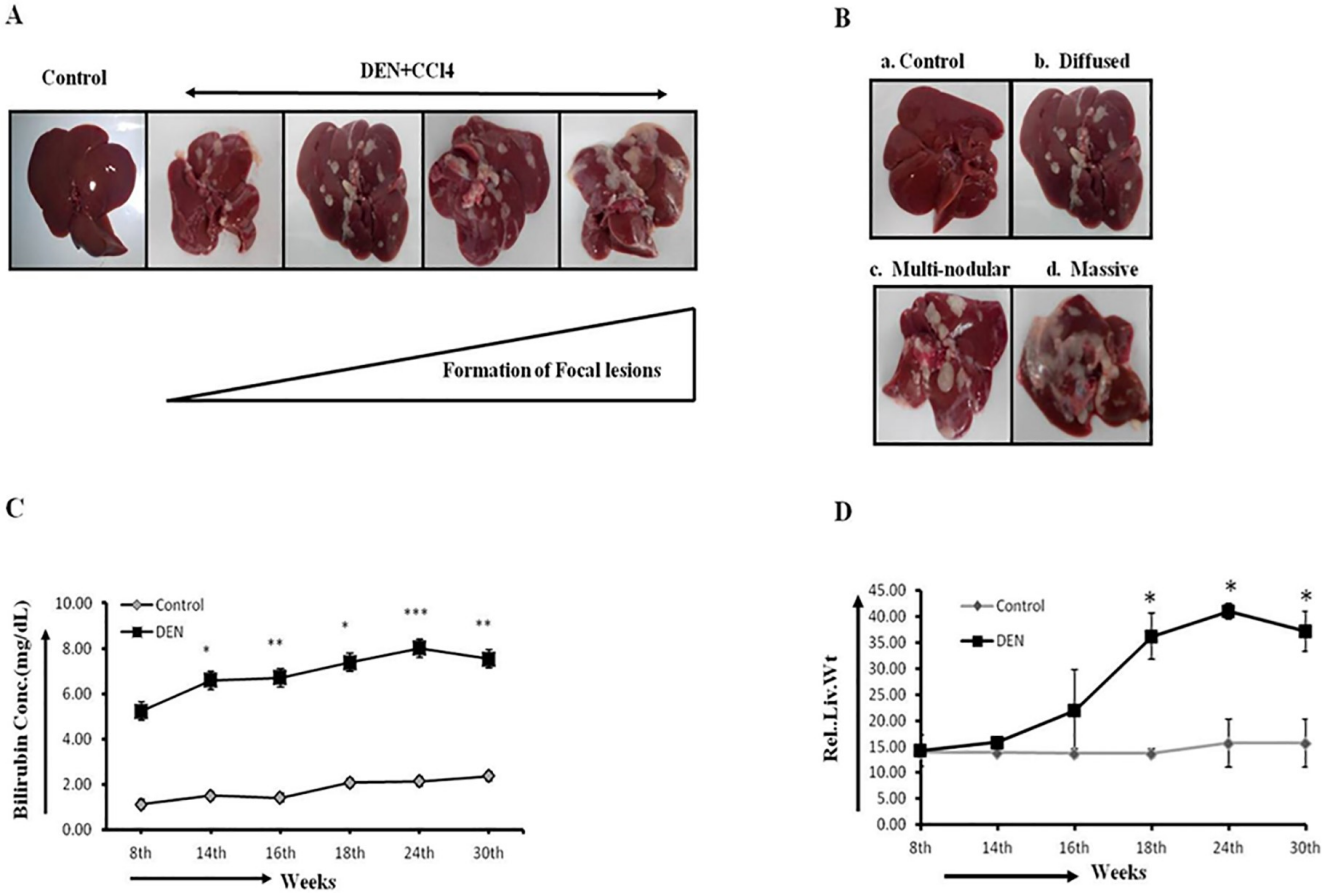
DEN induced HCC is associated with expanding nodular pattern during hepatocarcinogenesis. The expanding nodular pattern of HCC in male wistar rats. (A) Formation of focal lesions in DEN+CCl4 treated rats. Animals sacrificed at different stages of hepatocarcinogenesis. (B) The different patterns of nodular lesions observed in DEN+CCl4 treated animals- control (a); diffused (b), multi-nodular (c), and massive (d). (C) Estimation of bilirubin showing constantly increasing level in DEN+CCl4 treated animals. (D) Measurement of liver weight showing increasing trend at different time points in the treated group, which belonged to different stages of hepatocarcinogenesis. Data presented are expressed as Mean±S.D. *p<0.05, **p<0.005, ***p<0.0005.

Chemical induced rodent model mimics the injury-fibrosis-malignancy pattern following treatment with a genotoxic chemical alone or in combination with a promoting-agent[17]. DEN is a potent hepatocarcinogen and is widely used for experimental hepatocarcinogenesis in rodent models (reviewed in [18]). The DEN+Carbon tetrachloride (CCl4) model of rat hepatocarcinogenesis was followed[19–20] in our experiment. At the completion of the 8‑week treatments, and after latency-period of two weeks we observed altered hepatic foci or morphologically recognizable lesions in DEN treated animals. The size and number of nodules increased with time (Fig 1A) which showed the evidence of multi-nodular tumor in liver. The early phase was associated with smaller and lesser number of nodules while the late phase was associated with big nodular, massive and diffused type of appearances in different stages of hepaotocarcinogenesis. The most common nodular type included multiple nodules, massive type included large tumor with irregular demarcation, and the diffused type included many small nodules in whole liver (Fig 1B). The expanding nodular pattern was simultaneously associated with increase in bilirubin concentration in serum and increase in relative liver-weight (Fig 1C and 1D). We also found a strong positive correlation between liver-weight and body-weight of each animal in each group (S1A–S1D Fig). Remarkably, the R^2/PE value was maximum in the promotion-group.

### Early HCC show increased cell proliferation along with β-catenin (CTNNB1) activation

**Fig 2:**
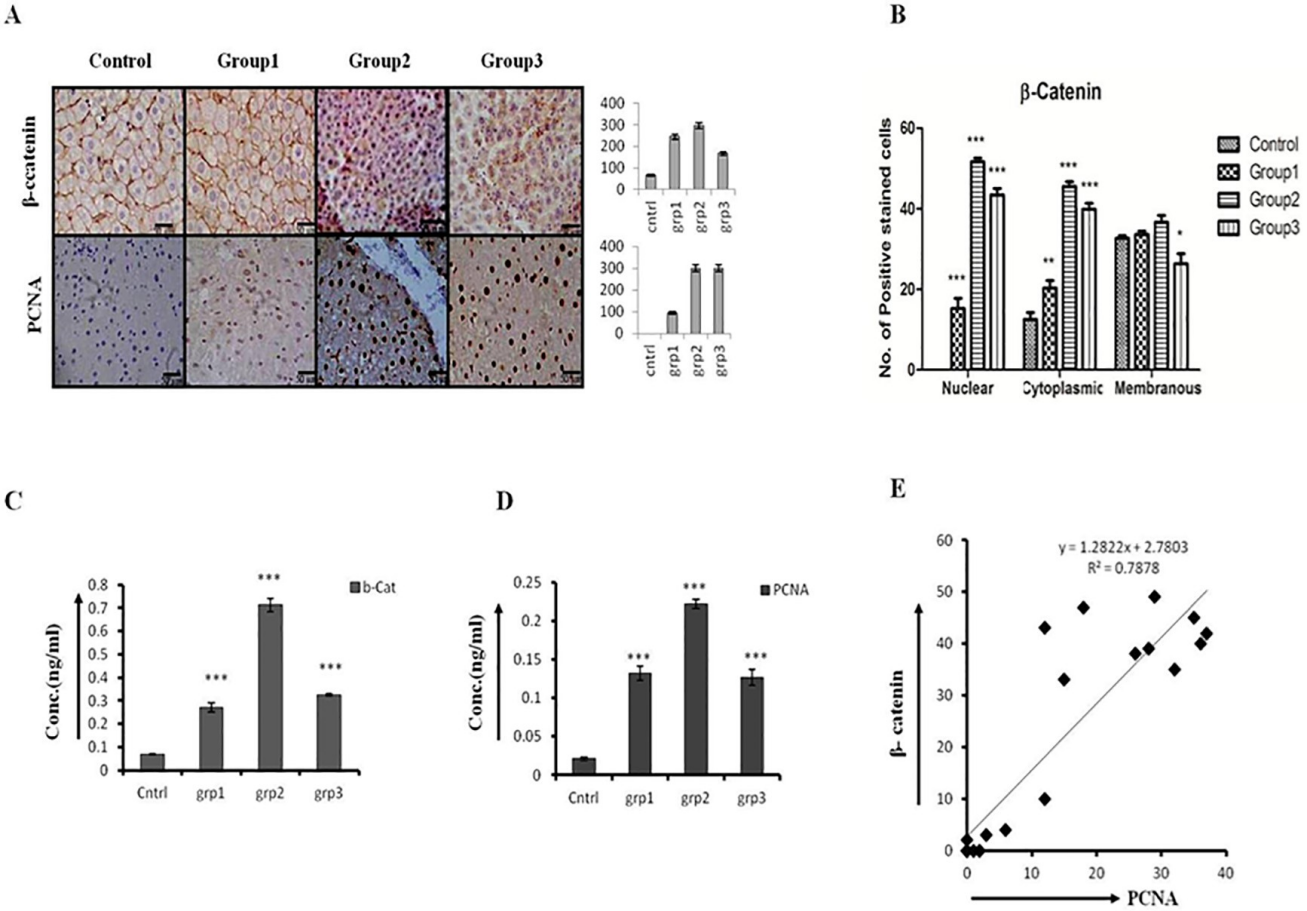
Early HCC show increased cell proliferation along with β-catenin (CTNNB1) activation. Expression levels of β-catenin and PCNA across all stages of hepatocarcinigenesis. (A) IHC staining of β-catenin and PCNA in rat liver tissue sections of initiation (Group1), promotion (Group2), and progression (Group3) stages of hepatocarcinogenesis. The corresponding IHC staining intensity graph is shown in the side panel of figures.Y-axis represents labeling index (%) visual score in each intensity graph. (B) Graph showing the number of positive stained cells for nuclear, cytoplasmic, and membranous staining of β-catenin. The number of positive cells were counted belonging to five different fields of five different sections of control, group1, group2 and group3 animals. (C) & (D) Quantitation of β-catenin and PCNA concentration in rat tissue lysates of different groups by ELISA. (E) Graph showing a strong positive correlation between β-catenin and PCNA across all stages of hepatocarcinogenesis. Data presented are representative of three independent experiments performed in triplicates and expressed as Mean±S.D. *, ** and *** differs significantly at *p* <0.05,<0.005 and <0.0005.

We next checked the expression level of β-catenin, which increased significantly in initiation- and promotion-group followed by a modest decrease in progression-group as compared to promotion-group (Fig 2A, upper panel). The increased β-catenin expression correlates with the increasing level of PCNA staining (Fig 2A, lower panel). The cytonuclear expression of β-catenin was more in promotion and progression group animals as evident by the quantification of positively stained cells (Fig 2B). The decrease in β-catenin and PCNA in progression-group is more evident in ELISA of tissue lysates (Fig 2C and 2D). Though the promotion-group animals showed more intense nucleo-cytoplasmic staining for β-catenin, the overall intensity got decreased in progression-group animals as demonstrated by intensity-score graph (Fig 2A side panel). There is strong positive correlation between β-catenin and PCNA staining among all three groups of hepatocarcinogenesis (Fig 2E).

### Higher level of Gli1/2 in late/progressed HCC coincide with an increase in sFRP1

**Fig 3:**
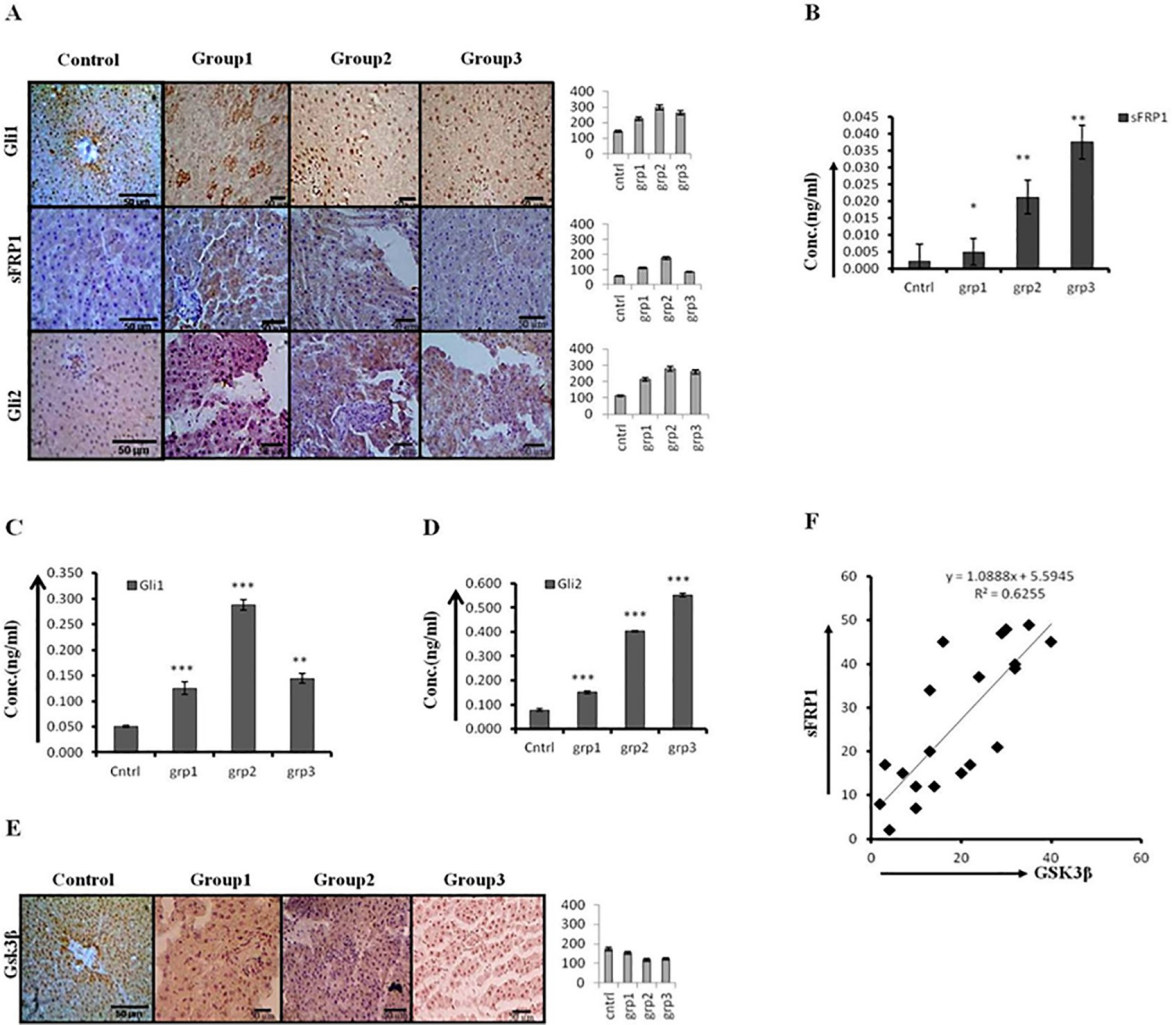
Higher level of Gli1/2 in late/progressed HCC coincide with an increase in sFRP1. Enhanced expressions of Gli1, Gli2, and sFRP1 at promotion stage. (A) IHC staining of Gli1, Gli2, and sFRP1 in rat liver tissue sections of initiation (Group1), promotion (Group2), and progression (Group3) stages of hepatocarcinogenesis. The corresponding IHC staining intensity graph is shown in the side panel of figures. Y-axis represents labeling index (%) visual score in each intensity graph. (B) Quantitation of sFRP1 in the serum of animals of different groups as estimated by ELISA. (C) & (D) Quantitation of Gli1 and Gli2 in rat tissue lysates of different groups by ELISA. (E) IHC staining of GSK3β in different groups of animals (left) and the corresponding IHC staining intensity graph (right), Y-axis represents labeling index (%) visual score in each intensity graph. (F) Graph showing a strong positive correlation between sFRP1 and GSK3β across all stages of hepatocarcinogenesis. Data presented are representative of three independent experiments performed in triplicates and expressed as Mean±S.D. *, ** and *** differs significantly at *p* <0.05,<0.005 and <0.0005.

The expression level of Gli1/2 was modest during early phase of HCC but it increased significantly in promotion-group and sustained through the progression-group animals. The quantification of Gli1 nuclear staining demonstrated maximum staining in promotion group animals (S2A Fig). This sustained increase in Gli1 in progression-group is associated with a corresponding increase in sFRP1, an inhibitor of Wnt pathway (Fig 3A). Although the increase in sFRP1 level is modest at protein-level in tissue sections ((Fig 3A) and in tissue lysates (S2B Fig), but as it is a secretory molecule so we checked its expression level in serum (Fig 3B). Interestingly, the level of sFRP1 in serum of progression-group animals was significantly higher as compared to initiation- and promotion-group animals. It has been already reported that Gli1 inhibits Wnt pathway via. direct transcriptional control of sFRP1[21]. We next checked the expression level of GSK3β as it is known to regulate Wnt β-catenin signaling and is in-turn controlled by sFRP1 [22]. The expression level of GSK3β was slightly decreased in the promotion-group animals (Fig 3E), the same group is associated with maximum Wnt and Hh activity. We found a strong positive correlation between sFRP1 and GSK3β (Fig 3F).

### The increasing Wnt signaling in initiation-group correlates with well-differentiated/grade I HCC

**Fig 4:**
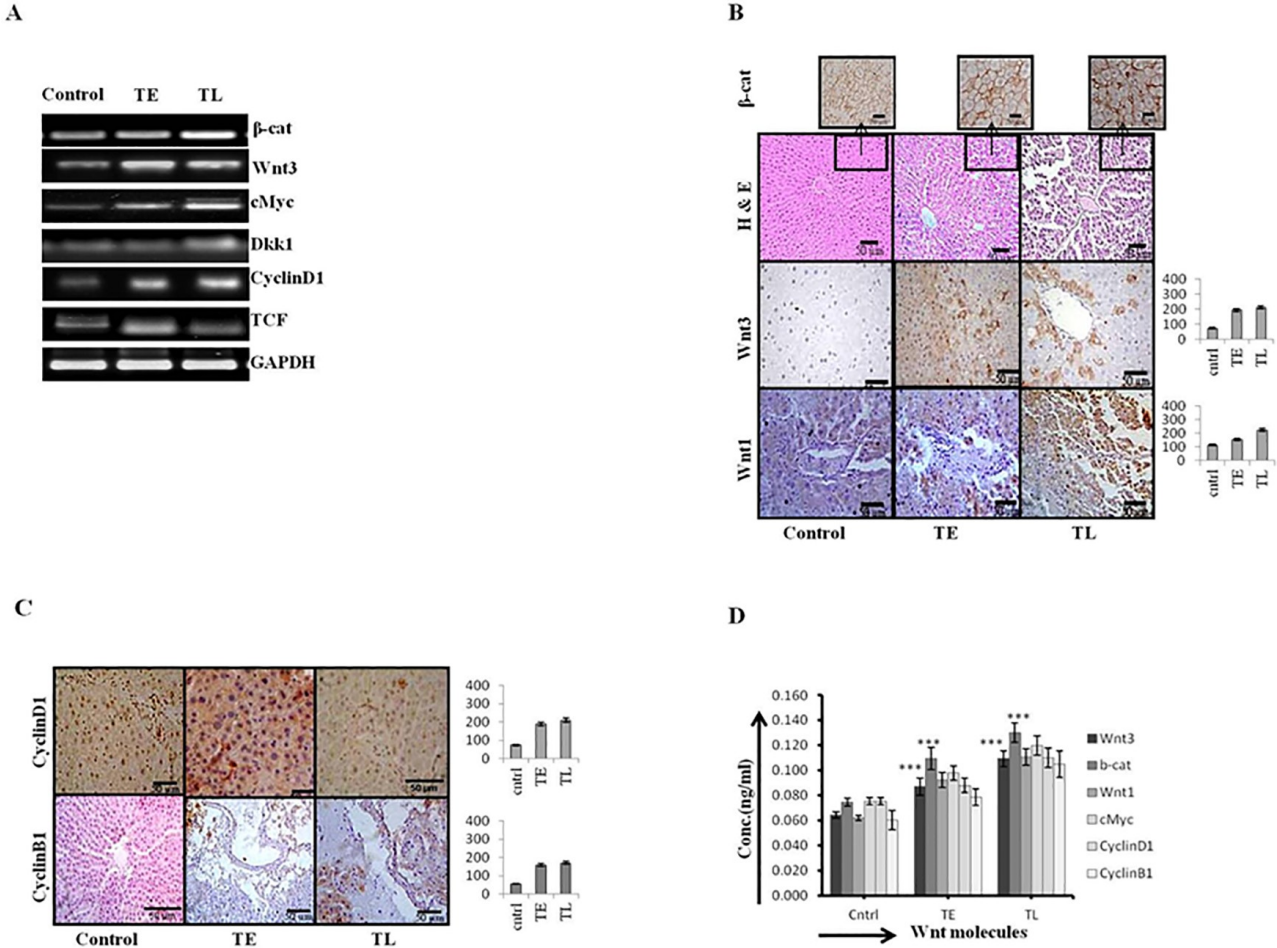
The increasing Wnt signaling in initiation-group correlates with well-differentiated/grade I HCC. Increased Wnt signaling correlates with well differentiated HCC. (A) Increased mRNA expression level of downstream Wnt signaling pathway of control and initiation group (group1) animals. The group1 animals sacrificed at 2 different time points early and late (TE and TL) separated by an interval of one week. (B) H and E stained liver sections of control and group1 animals showing well differentiated, thin trabecular pattern at the upper panel, IHC images of β-catenin staining of the similar section is shown as the top projection. (C) IHC staining of two Wnt pathway target genes, cyclin D1 and B1 in group1 at TE and TL phases of initiation stage and the corresponding IHC intensity graph. Y-axis represents labeling index (%) visual score in each intensity graph. (D) Analysis of Wnt pathway molecules in rat tissue lysates at TE and TL phases of initiation stage by ELISA method. Data presented are representative of three independent experiments performed in triplicates and expressed as Mean±S.D. *** represents *p- value* <0.0005.

HCC is a hyper vascular tumor with various differentiation status and histological-patterns. Among different histological-pattern the trabecular-pattern is the most common which is associated with both well-differentiated and moderately-differentiated HCC. The thickness of hepatic plates differs in both cases as of 2-cells thick in well-differentiated to 4-cells thick in moderately-differentiated HCC. During the initiation stage the increasing expression of β-catenin, Wnt-1, Wnt-3, Cyclin D1, DKK1, and c-Myc were observed at mRNA (Fig 4A) and protein level by IHC staining (Fig 4B and 4C) and by ELISA for tissue lysates (Fig 4D). The corresponding change in the histological properties is shown in Fig 4B upper panel. Results demonstrate the well-differentiated morphology of HCC with thin trabecular-pattern and corresponding increased level of Wnt pathway molecules suggesting the activation of Wnt pathway is associated with early well-differentiated pattern of HCC.

### The higher activity of Wnt and Hh pathway in promotion-group correlates with moderately-differentiated/grade II HCC

**Fig 5:**
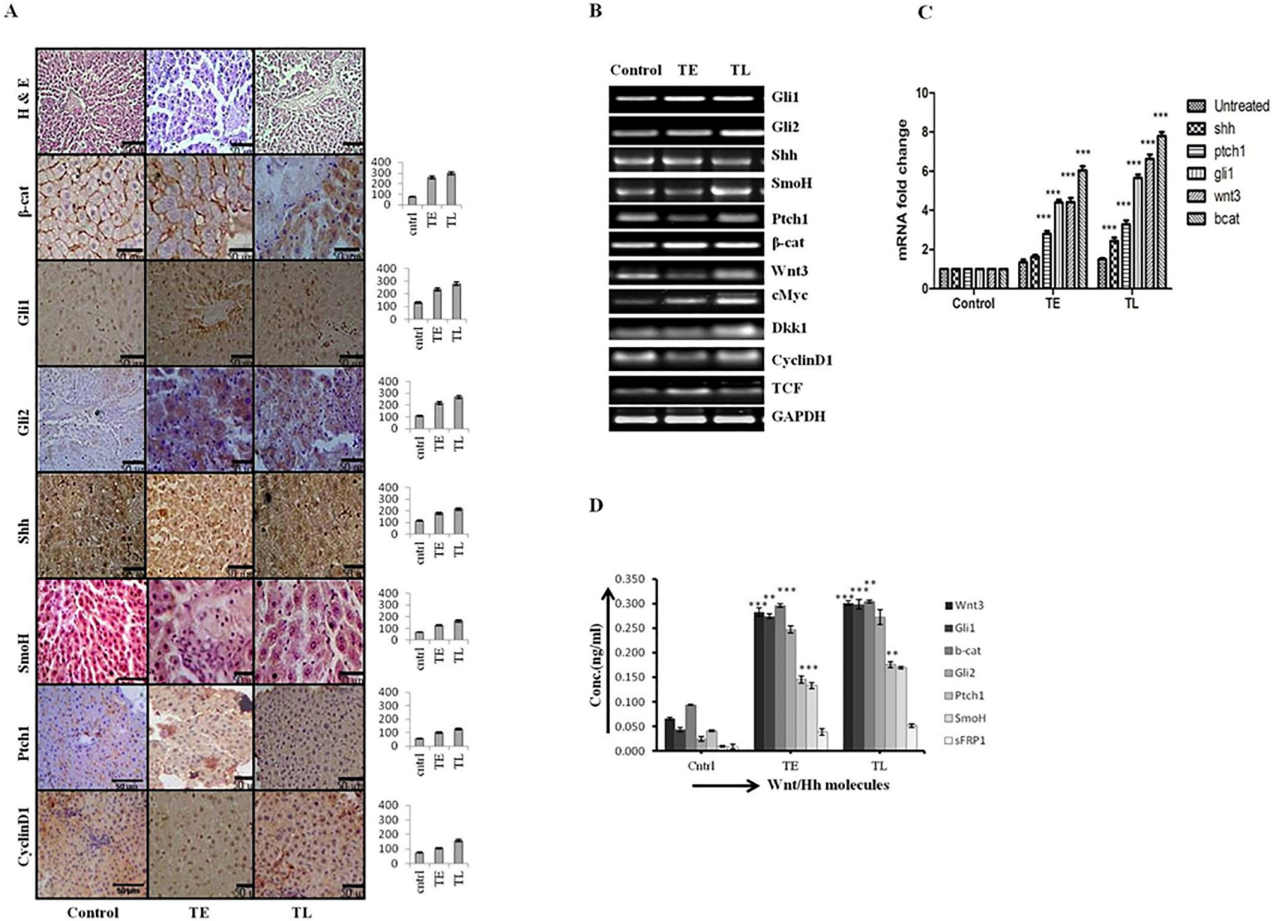
The higher activity of Wnt and Hh pathway in promotion-group correlates with moderately-differentiated/grade-II HCC. Enhanced Wnt and Hh signaling correlates with moderately differentiated HCC. (A) H and E stained liver sections of control and promotion group (group2) animals showing moderately differentiated, thick trabecular pattern, followed by photomicraphs showing IHC staining of Wnt and Hh pathway molecules at TE and TL phases of promotion stage. The corresponding IHC staining intensity graph is shown in the side panel of figures.Y-axis represents labeling index (%) visual score in each intensity graph. (B) Increasing mRNA expression level of Wnt and Hh pathway molecules of control and group2 animals at TE and TL phases of promotion stage. (C) The figure represents fold increase in relative mRNA expression, calculated with respect to GAPDH for Shh, Ptch1, Gli1, Wnt3 β-catenin in control and group2 animals at TE and TL phases of promotion stage (D) Analysis of Wnt and Hh pathway molecules in rat tissue lysates at TE and TL phases of promotion stage by ELISA. Data presented are representative of three independent experiments performed in triplicates and expressed as Mean±S.D. ** and *** differs significantly at *p* <0.005 and <0.0005.

Our results demonstrate that activation of Wnt signaling pathway is an early event in hepatocarcinogenesis. While the Hh pathway was activated little later than Wnt pathway, the expression of Wnt3/3A, β-catenin, Gli1, Gli2, Ptch1, Smo, were maximum during the promotion stage of hepatocarcinogenesis as evident by our IHC, RT-PCR, qRT-PCR (Primers used for RT-PCR and qRT-PCR are listed in Table 1), and ELISA results (Fig 5A–5D) respectively. The IHC staining intensity quantification demonstrated maximum staining in promotion-group animals as compared to other groups. These results are complemented with the ELISA readouts showing maximum expression level of these molecules in promotion-group animals. The increased activation of Wnt and Hh pathway correlates well with the moderately-differentiated histological pattern of HCC (Fig 5A, upper panel).

**Table 1:** Primer set of various genes adopted in RT-PCR.

Genes	Sense	Anti-Sense	Annealing Temp.	No. of Cycles
β-catenin	AACGGCTTTCGGTTGAGCTG	TGGCGATATCCAAGGGCTTC	60°C	30
Wnt3	GGAGAAACGGAAGGAGAAATG	GAGAGACGTTAGTTGAGAAAGAAGC	58°C	30
cMyc	CCAGGACTGTATGTGGAGCG	CCTGAGGACCAGTGGGCTGT	56.6°C	34
Gli1	GGGATGATCCCACATCCTCAGTC	CTGGAGCAGCCCCCCCAGT	56.6°C	34
Gli2	GCAGGTGTATCCCACGGAAAGCACTG	CTCTCCTCGGCGAGGCTGGTGAGCAT	57.9°C	30
COX2	TTCAAATGAGATTGTGGGAAAAT	AGATCATCTGCCTGAGATATCTT	57°C	32
Shh	GATGTCTGCTGCTAGTCCTCG	CACCTCTGAGTCATCAGCCTG	55.3°C	32
SmoH	GTTCTCCATCAAGAGCAACCAC	CGATTCTTGATCTCACAGTCAGG	55.3°C	32
CyclinD1	CTGGCCATGAACTACCTGGA	GTCACACTTGATCACTCTCC	54.3°C	30
Ptch1	CAGAGAAGGCTTGTGGCCAC	GCTCAATGACTTCCACCTTCG	56.2°C	32
DKK1	ATTCCAACGCTATCAAGAACC	CCAAGGTGCTATGATCATTACC	55°C	30
TCF	TGACCTCTCTGGCTTCTACT	TTGATGGTTGCTTCTTGGC	53°C	32
GAPDH	ATCTTCCAGGAGCGAGATCCC	CGTTCGGCTCAGGGATGACCT	58°C	30

### The decreasing Wnt signaling and sustained Hh signaling correlates with poorly-differentiated/grade III HCC

**Fig 6:**
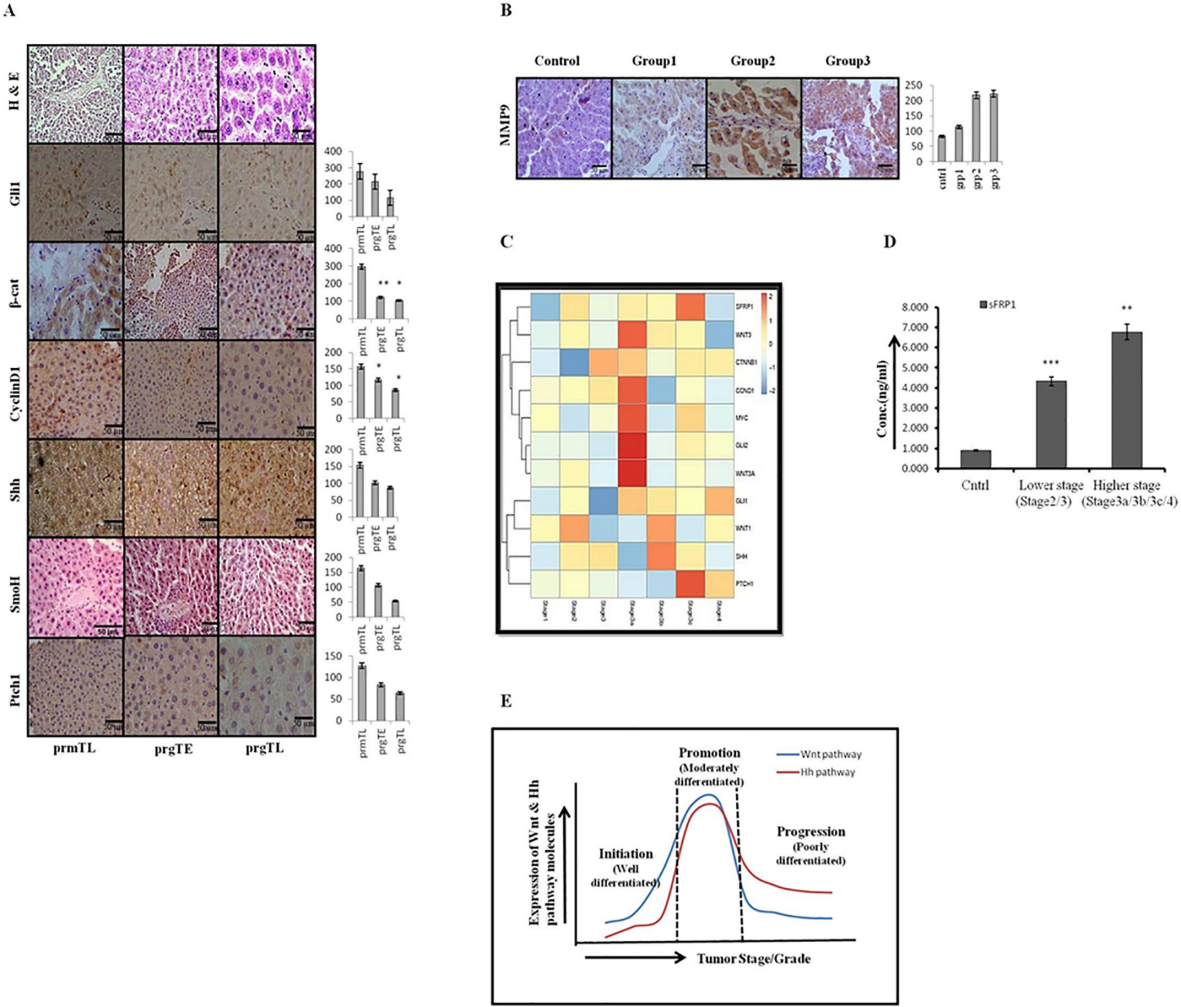
The decreasing Wnt signaling and sustained Hh signaling correlates with poorly-differentiated/grade-III HCC. Sustained Shh signaling correlates with poorly differentiated HCC. (A) H and E stained liver sections of promotion (group2: prm TL) and progression group (group3: prgTE and prgTL) animals showing poorly differentiated, thick solid pattern, followed by photomicraphs showing IHC staining of Wnt and Hh pathway molecules at TE and TL phases of progression stage. The corresponding IHC staining intensity graph is shown in the side panel of figures. Y-axis represents labeling index (%) visual score in each intensity graph. (B) IHC staining of MMP9 in Group1, 2, and 3 animals showing increased expression. (C) The heatmap of RNASeq data showing expression level of different Wnt and Hh pathway genes with the HCC stages/grade. (D) Serological analysis for sFRP1 in patients samples of lower and higher stages of HCC. (E) Conclusive figure showing the trend of Wnt and Hh pathway molecules expression in correlation to the histological grade/stage of HCC. Data presented are representative of three independent experiments performed in triplicates and expressed as Mean±S.D. ** and *** differs significantly at *p* <0.005 and <0.0005.

We next examined the expression level of Wnt and Hh pathway molecules in progression-group animals. As evident by our results the expression level of Wnt3/3A and β-catenin got modestly decreased in the progression-group animals (Fig 6A and S3A and S3B Fig) while the expression level of Shh, Gli1/2, and Ptch1 was consistently high in progression-group animals. Although, there was decrease in Hh pathway molecules in the later phase (TL) of progression-group, the decrease was not significant as demonstrated by the intensity-score graph. The sustained expression of Hh and decreased expression of Wnt pathway molecules correlate with poorly-differentiated/solid histological-pattern of HCC (Fig 6A upper panel). We also checked the immunostaining intensity of MMP-9 in all three groups of animals. Our results demonstrated increased cytoplasmic expression of MMP-9 in group2 and 3 animals (Fig 6B and S3C Fig).

### Up-regulation of sFRP1 in higher stage HCC patient's biospecimens

In order to test the translational value of our *in vivo* data into clinical application we performed the expression analysis of Wnt and Hh pathway molecules in HCC patients of different stages available at TCGA data-base. As the TCGA data-base provides large scale genomic datasets for different type of cancer including HCC, we used these patient’s data, to corroborate our results from the animal model. These comparisons indicate that to a large extent animal model observations are supported by the patient’s biospecimen data. The results demonstrated relatively higher level of expression of Gli1 and sFRP1 in intermediate and advanced stage HCC, as shown by heat-map (Fig 6C). It is also evident by our analysis that the expression levels of WNT3/3a, β-CATENIN, C-MYC, GLI2, PTCH1, SHH were maximum during the stage3a and 3b of HCC patients (Fig 6C), which corroborates our *in vivo* findings. Additionally, we also obtained blood samples from 6 healthy and 12 HCC patients of different stages (patient details are summarized in Table 2) and performed the serological analysis of sFRP1. The results demonstrated increased level of sFRP1 in higher stage patients (Fig 6D) as compared to the lower stage patients, which is in support of our *in vivo* findings.

**Table 2:** Patients details with HCC grade/stages.

Serial No.	Age	Sex	Stage/Grade
1	72	Male	Stage-4
2	72	Female	Stage-2
3	50	Male	Stage-2
4	35	Male	Stage-3c
5	80	Male	Stage-3
6	57	Male	Stage-2
7	70	Male	Stage-2
8	63	Male	Stage-3
9	56	Female	Stahe-3c
10	76	Male	Stage-3
11	51	Female	Stage-3c
12	73	Male	Stage-3a/3b

## Discussion

The significance of the morphological lesions and their histopathological appearance should be meaningfully conveyed to the clinicians. The purpose of grading and staging in cancer diagnosis is to record those histomorphic abnormalities which are responsible for the severity and progression of the disease. Depending upon the quality and size of biopsies available the semi-quantitative grading and staging system should be considered in approximation so it should be better correlated to the molecular signatures of the tumors. Both grading and staging of cancers have prognostic significance for many cancers including HCC, but in case of HCC, there is a significant degree of heterogeneity in its microscopic assessment [23]. Therefore we need a more defined way to assess the microscopic features of HCC. In the present study, we explored whether Wnt and Hh pathway gene expression profile could be used to grade and stage HCC more precisely.

Our DEN-induced hepatocarcinogenesis model in Wistar rats resulted in 100% tumor incidence, small focal nodules during early phases and increased bilirubin level in DEN-treated animals revealing liver function alteration which is the first sign of diseased liver including carcinogenesis. The strong positive correlation between liver-weight and body-weight of each animal in each group suggested that active regeneration is taking place after DEN-CCl4 treatment. Notably, the R^2/PE value was maximum in the promotion-group which is associated with the maximum activity of Wnt and Hh pathway and consequently and genes involved in cell-proliferation were active during this stage.

As hepatocarcinogenesis is a multistep process which involves various molecular changes and associated phenotypes, so we performed expression analysis of various Wnt and Hh pathway molecules during the initiation, promotion, and progression stages. Our results demonstrated the membranous expression of β-catenin in the initiation-group animals was feebly increased as compared to the control. There was more nuclear expression of β-catenin in promotion-group which further increased in progression-group animals (Fig 2A and 2B), these results uphold the previously published work by Suzuki et al., (2002) [24]. The cytoplasmic expression of β-catenin was modest and only minor nuclear expression was found in initiation-group (Fig 2B). Our data demonstrates that total β-catenin is getting reduced in progression group but overall nuclear expression of β-catenin is increased in the same group (Fig 2B). We examined cell-proliferation at different stages of DEN induced hepatocarcinogenesis by investigating the PCNA expression level (Fig 2A and 2D). We found a very strong positive correlation between β-catenin and PCNA expression among all group of animals (Fig 2E) which suggest β-catenin plays important role in tumor promotion by stimulating tumor cell-proliferation. Furthermore, the nuclear expression of β-catenin was more in the progression-group animals, and it is already reported that nuclear expression of β-catenin is associated with invasiveness and metastasis in colorectal carcinoma[25]. Additionally, Inagawa et al.,(2002) demonstrated that nuclear accumulation of β-catenin in HCC is associated with tumor progression and poor prognosis, particularly in poorly-differentiated HCC patients[26].

Our results demonstrate that the expression level of Gli1, which is a marker for Hh pathway activation, was modest in the initiation-group and was increased significantly in the promotion-group animals particularly the nuclear expression of Gli1 was more in promotion group (S2A Fig). The increase in Gli1/2 was associated with a similar increase in sFRP1, an inhibitor of Wnt pathway (Fig 3A and S2B Fig). Although the expression level of sFRP1 was not much in tissue-sections of promotion-group and decreased in the progression-group as demonstrated by IHC staining of liver tissue-section (Fig 3A) and ELISA of tissue-lysates (S2B Fig). But the level of sFRP1 was significantly more in the blood serum of promotion- and progression-group (Fig 3B) which suggest the importance of secreted form. He et al., (2006) reported that Gli1 transcriptionally activate sFRP1 which in turn inhibits Wnt signaling pathway[21], suggesting the regulation of Wnt signaling pathway by Hh signaling pathway. However, in our experimental model it is likely that sFRP1 only modestly affect β-catenin /Wnt signaling pathway. sFRP1 is known to be expressed as cytoplasmic, extracellular, and secreted form. Although in many cancers sFRP1 is inactivated due to epigenetic modification [27–28], but it is also known to be over-expressed in gastric cancer and renal cell carcinoma [29–30] where it is associated with tumor aggressiveness, metastasis, and decreased overall survival. It has been reported that sFRP1 over-expression in gastric cancer cells correlates with the activation of TGF β-catenin pathway, which is in-turn responsible for the invasiveness of cancer[29]. Noticeably it has been demonstrated by Qu et al., (2013) that sFRP1 can be used as a biomarker for aggressive gastric cancer [29].

The sFRP1 expression is known to activate GSK3β through different pathways [22]. GSK3β is a key component of various signaling pathways including Wnt and Hh, and is known to negatively regulate both pathways. Our data demonstrated a strong positive correlation between sFRP1 and GSK3β expression among all stages of hepatocarcinogenesis (Fig 3F).

The activation of β-catenin as an early event in hepatocarcinogenesis is already reported [31]. The β-catenin expression and role have been studied in a number of cancers including HCC but it has not been studied in the context of histopathological heterogeneity of cancers. In our results the Wnt pathway was activated in the early phase of HCC initiation (TE) although it was a modest activation which later became significant in the later-initiation (TL) phase. The Wnt target genes also got activated in the similar way suggesting the role of Wnt pathway in the initiation stage of hepatocarcinogenesis (Fig 4A–4D). Increased protein expression in the early phase is due to increased transcription as evident by the increase in mRNA level of Wnt pathway molecules (Fig 4A). The activation of Wnt signaling correlates adequately with the well-differentiated histological-pattern of HCC (Fig 4B). The target genes of Wnt pathway like cyclin D1, cyclin B1, c-Myc significantly increased during the late-initiation (TL) phase (Fig 4C and 4D). These findings suggest that Wnt pathway activated during the initiation stage of DEN-induced hepatocarcinogenesis in Wistar rats and correlates with the well-differentiated histological-pattern of HCC.

The Hh activity significantly increased during the early promotion stage of hepatocarcinogenesis which is simultaneously associated with increased expression and activity of Wnt pathway (Fig 5A–5C). All the molecules of Wnt and Hh pathways demonstrated maximum level of expression during the promotion stage of hepatocarcinogenesis, as evident by our IHC, ELISA, and qRT-PCR results (Primers listed in Table 1). Higher expression and activity of both Wnt and Hh pathways were associated with a thick trabecular-pattern which corresponds to the moderately-differentiated HCC (Fig 5A). On the other hand the progression-group animals were associated with a modest decrease in Wnt activity in TE phase which was more significant during the TL phase (Fig 6A, S3A and S3B Fig). The increased Wnt and Hh pathway activity during promotion stage were simultaneously associated with an increase in expression of MMP9 (Fig 6B), which sustained until progression stage although it got reduced in serum samples of progression-group animals as demonstrated by our ELISA data (S3C Fig). In HCC MMP9 expression and activity is predictive of metastatic and invasive carcinoma [32]. Our results demonstrated increased expression of MMP-9 from initiation through progression stage of hepatocarcinogenesis, which is in agreement with the previously published report[33]. Furthermore, our data demonstrated that during the progression stage Wnt pathway expression and activity decreased modestly as compared to promotion stage, but the Hh pathway expression and activity sustained. Moreover, the sustained activity of Hh pathway is associated with the aggressive and invasive phenotype of HCC as evident by our *in vitro* results in HCC cell-line Hep3B. After treatment with purmorphamine, an activator of Hh pathway, the migration of cells increased (S4 Fig). Several studies have shown that Hh signaling is involved in regulating motility of various cell-types [34–35]. Feldmann et al., (2007) reported that inhibition of Hh signaling hinders pancreatic cancer invasion and metastasis [36]. Furthermore, the role of Hh signaling pathway in proliferation and invasiveness of HCC cells has also been reported[37]. All these studies support our finding that sustained activation of Hh pathway is associated with progression stage of hepatocarcinogenesis as well is coupled with the solid histological-pattern and poorly-differentiated histopathology(Fig 6A upper panel). On the other hand the β-catenin activity increased during the progression stage of hepatocarcinogenesis post LiCl treatment in DEN+CCL4 treated rats as evident by our IHC result(S5 Fig). LiCl is a known activator of Wnt pathway which increased β-catenin expression by inhibiting GSK3β [38–39]. Higher expression and activity of β-catenin was associated with a thin trabecular-pattern which corresponds to the well-differentiated histological pattern of HCC instead of moderately or poorly-differentiated histological pattern.

Furthermore, the expression analysis of Wnt and Hh pathway genes in HCC patients’ samples available at TCGA data-base supported our results and facilitated the molecular understanding of HCC histopathology. The heat-map showing expression level of different Wnt and Hh pathway genes revealed the higher expression level of most of the Wnt pathway genes was during the early stages of hepatocarcinogenesis (started from stage 3), while the expression level of Hh pathway genes were more during the intermediate and late stages (stage 3a and above) as demonstrated in Fig 6C. We observed high level of Gli1/2 above stage 3a and significantly high level of sRFP1 in stage 3c patients, which is consistent with our *in vivo* data representing increased level of sFRP1 in progression-group animals. These results were also corroborated by the serological analysis of sFRP1 in blood-samples of HCC patients of different stages, demonstrating higher level of sFRP1 in higher stage patients (Fig 6D) which included patients of stage 3a and above. There are few reports which indicate the hypermethylation of sFRP1 promoter in HCC which is responsible for its silencing[40–41], but most of these reports deals with the promoter methylation and expression level of sFRP1 in HCC cell-lines. Moreover, Shih et al., (2006) demonstrated the extent of sFRP1 promoter methylation in HCC cell-lines and patients samples. They reported that sFRP1 inactivation due to promoter methylation was predominantly above 65% in HCC cell-lines compared to the HCC patients samples which were predominantly below 48%[42]. Furthermore, they also demonstrated that sFRP1 promoter methylation decreased significantly compared to the HCC patients (48.2%), than in patients with cirrhotic liver (21.4%) and chronic hepatitis (14.3%). These results infer that the extent of sFRP1 promoter methylation and inactivation in HCC patients is highly dependents on the epidemiology of HCC. Besides, Davaadorj et al., (2016) demonstrated that a higher expression of sFRP1 in HCC patients significantly correlated with higher age group of patients, with most of the patients above 65 years of age expressing higher level of sFRP1[43]. Interestingly, we also observed that most of the patients who were above 65 years old were associated with higher level of sFRP1 (Table 2), although one of the limitation of our research was working with small number of patients. Furthermore, the high level of sFRP1 in higher stage HCC patients could be used as a potential biomarker for HCC. Furthermore, sFRP1 has been demonstrated to be a potential biomarker in breast cancer survivors[44] which support the notion that serological sFRP1 could be a potential biomarker for HCC.

Advanced HCC could be either moderately-differentiated or poorly-differentiated but clinically these two cases have always been treated inseparably. The outcome of curative treatments like surgical resection and liver transplantation for poorly-differentiated HCC patients was worse than the moderately- or well-differentiated HCC patients as described Jonas et al., 2001[45]. So for a better clinical outcome, the molecular differentiation of these two cases of advanced HCC is of utmost importance. Besides, it is already known that morphologically similar HCC can show different therapeutic response, which might be due to different molecular signatures. So integration of information regarding expression level of Wnt and Hh pathway molecules during grading and staging of HCC might improve its stratification for the selection of available therapeutic strategy.

Conclusively, our data uncover the grade-related expression of Wnt and Hh pathway molecules which is simultaneously associated with a shift in histological pattern from well-differentiated to poorly-differentiated (Fig 6E). The dynamic natures of HCC histopathology must be taken into consideration before deciding the stage specific treatment plan. Although, the heterogeneous nature of HCC is well documented but its role in histopathology and allied molecular connection was not thoroughly investigated. Wnt and Hh pathway genes differentially/distinctly expressed in lower/higher stage/grade have a potential utility for treatment of HCC patients based on tumor characteristics and behavior.

## Materials and methods

### Reagents and antibodies

N-Nitrosodiethylamine (DEN, Cat:N0258), GoTaq Green Master Mix (Cat:M712B) were purchased from Sigma Aldrich and Promega respectively. TRIzol reagent (Cat:15596026), Verso c-DNA synthesis kit (Cat:AB1453A) were purchased from Thermo Fisher Scientific, Inc.(USA). Primary antibodies β-Catenin (Cat: SC-7963), Wnt3 (Cat: ab50341), PCNA (Cat: SC-56), Gli1 (Cat: SC-20687), Gli2 (Cat: SC-28674), sFRP1 (Cat: ab126613), MMP9 (Cat: SC-393859), GSK3β (Cat: SC-9166), Shh (Cat: SC-9024), CyclinD1(Cat:SC-20044), CyclinB1(Cat:SC-752) were purchased from Santa Cruz Biotechnology (Santa Cruz, CA). A horseradish peroxidase (HRP) conjugated goat anti- rabbit IgG, goat anti-mouse IgG secondary antibodies (Cat: ab97200 and ab97265 respectively), Rabbit Specific HRP/DAB (ABC-IHC) Kit (Cat: ab64261) were purchased from Abcam.

### *In vivo* experiments and Hepatocarcinogenesis model

*In vivo* studies were performed with male Albino Wistar rats of 120-150g body weight, which were procured from the laboratory animal facility of KIIT School of Biotechnology. They were housed in standard temperature/ humidity conditions and environment (12hr light/dark cycle). All animals were provided standard pellet diet and water ad libitum. All the experimental protocols were approved by the Institutional Animal Ethics Committee (IAEC, KIIT School of Biotechnology, Bhubaneswar, India).

The rats were randomly and evenly allocated into four groups, six rats in each group. All animals were acclimatized for two weeks before starting the experiment. The DEN + CCl4 model of rat hepatocarcinogenesis was followed in our experiment. N-Nitrosodiethylamine (DEN) was administered in group 1, 2, and 3 as 100 mg/kg body weight per week for three consecutive weeks. After 1 week of recovery period, the promoting reagent CCl4 was given 2ml/kg body weight of rats weekly twice for consecutive 8 weeks. Group 1, 2, and 3 were assigned as initiation stage, promotion stage, and progression stage animals. At the completion of the 8 week treatments, and after latency period of two weeks we observed altered hepatic foci or morphologically recognizable lesions in DEN treated animals. The rats were sacrificed under anesthesia at two time points early (TE) and late (TL) for each group which were separated by the interval of one week to show the extent of histological pattern in each group. Before sacrifice the total food-water intake and body weights were recorded.

### RNA extraction and reverse transcription-quantitative polymerase chain reaction (RT-qPCR) analysis

Total RNA was extracted from the rat liver tissues with TRIzol reagent in accordance with the manufacturer’s instructions reverse transcription was performed in total volume of 20μl using 2μg of total RNA by Verso c-DNA synthesis kit. To quantify the changes in m-RNA level reverse transcription PCR and RT-qPCR were performed on the CFX Connect Real-Time PCR Detection System (Life-Technology, BIORAD). RT-PCR was performed by using GoTaq Green Master Mix. For this purpose β-catenin, Gli1, Gli2, Wnt3, Shh, SmoH, Ptch1, cMyc, CyclinD1, Cox2, Dkk1, TCF genes were used. PCR primers are designed based on human mRNA sequence. PCR products were separated by electrophoresis in 1% agarose gel, visualized by 0.5ug/ml ethidium bromide staining for 40mnts. The gel image and intensity of each band was measured by GEL-DOC Image software (BIO-RAD). q-PCR was performed by using SsoFast EvaGreen Supermix (BIORAD) with the following cycling conditions: 95°C for 5min, followed by 34 cycles of 95°C for 15sec and 60°C for 25sec. Glyceraldehyde 3-phosphate dehydrogenase (GAPDH) gene was used as the internal control. The primer details and cycles were described in Table 1.

### ELISA

The detection of serum samples collected from rats, whole cell lysates from rat liver tissues and human patient samples was done by Indirect ELISA method. Briefly, the protein antigen (1μg/ml conc.) was mixed with coating buffer (0.05 M) and coated onto 96 well microplate. The plate was then incubated overnight at 4°C followed by washing with 1X PBST and then blocking with1% BSA at room temperature for 2hrs. Then primary antibodies (1:2000dilution) were added into each well and incubated 2hrs at room temperature followed by washing thrice with 1X PBST. The wells were then incubated with secondary HRP linked antibody (1:2,500 and 1:5000 dilutions according to the primary antibody) for 45mints at room temperature. After washing thrice with 1X PBST, 2,2'-azino-bis (3-ethylbenzothiazoline-6-sulphonic acid) or ABTS substrate solution was added and absorbance was read at 410 nm using ELISA microtitreplate reader (EPOCH).

### Histopathology and Immunohistochemistry

- Histopathology analysis was done for the paraffin embedded tissue sections. The sections were deparaffinize and rehydrated in xylene followed by 100%, 95%, 80% ethanol and dH2O. Then the sections were stained by Hematoxylin and rinsed with dH2O to allow stain to develop. After that sections were dipped in acid-ethanol to destain and again rinsed with dH2O. Sections were stained with Eosin for 30sec, then dehydrated with 95%ethanol followed by 100% ethanol and xylene. Finally sections were mounted by coverslips with permount taking care to leave no bubbles and kept in the laminar hood to dry overnight. Imaging of the stained slides was done using Leica (DM 2000) bright-field microscopy. The pathological analysis of control and treated animals were done by evaluating both macroscopic and microscopic features of liver tissues. The slides were reviewed blindly by two pathologists and graded with respect to their histological characteristics as per WHO criteria for human and rat HCC [8,41].
- Immunohistochemistry analysis was done for tissue sections taken over poly-L-lysine coated slides. The tissue sections were taken from formalin fixed, paraffin embedded blocks. Sections were cut 4μm thick. Immunostaining was done by the use of Rabbit Specific HRP/DAB (ABC-IHC) Kit. The experiment was done according to the manufacturer’s protocol. Imaging of the stained slides was done using Leica (DM 2000) bright-field microscopy and the intensity score of each images was calculated by Immuno-Ratio Analysis software.

### HCC patients sample and TCGA data analysis

Blood samples from 6 control and 12 HCC patients of different stages were obtained from department of Gastroentrology & Hepatobiliary Sciences, IMS & SUM Hospital, Bhubaneswar, India. The blood samples were processed to obtain serum and were analyzed for sFRP1 expression level by Indirect ELISA method. The protocol was approved by Institutional Ethical Committee (IEC, Institute of Medical Sciences and SUM Hospital, S 'O' A University. Bhubaneswar, India) and written consent was taken by the patients. The information regarding patients age and stage is given in Table 2.

TCGA database was utilized for accessing HCC patients’ genomic data. As the TCGA data-base provides accessibility of large scale genomic datasets for analysis based on extensive co-operation, we courteously utilized the datasets. The TCGA LIHC datasets were analyzed consisting of maximum 10 patients in some stages of HCC. Not all stages had 10 patients as for some stages maximum available patient data was limited (Stage1: 10, Stage2: 10, Stage3: 6, Stage3a: 9, Stage3b: 8, Stage3c: 10, Stage4: 6).

### Statistical analysis

The data presented is the Mean±SD of three independent experiments. Changes in gene expressions and cytoplasmic-nuclear staining were analyzed by two way analysis of variance (ANOVA) using Grpah-Pad Prism5. The fold change of m-RNA was used as variables to compare samples between different treatment groups. Statistical analysis for ELISA readouts of tissue lysates were carried out using two tailed analysis,*p <0.05, **p <0.005, ***p <0.0005 were considered to be significant.

## Supporting information

S1 Fig Linear regression and extent of correlation was established between body weight and liver weight of animals of control, group1, group2, and group3. The graphs revealed that there is a significant correlation (R^2^/P.E>6) between liver weight and body weight in group1, 2, and 3 animals. To determine extent of correlation, probable error (PE) of coefficient of correlation was calculated with the formula PE = 0.6745(1-R^2^)/(6)1/2 (Fig 1C(a-d)). Panel (a) shows linear regression analysis graph for control, panel (b) for group1, panel (c) for group2, and panel (d) for group3 animals. These results depict considerable regeneration of liver taking place after DEN and CCl4 treatment.
(TIF)

S2 Fig Expression level of Gli1 with increasing sFRP1. (A) Graph showing the number of positive stained cells for nuclear, and cytoplasmic staining of Gli1. The number of positive cells were counted belonging to five different fields of five different sections of control, group1, group2 and group3 animals. (B) Expression level of sFRP1 in the rat tissue lysates of different groups (group1,2 & 3) with control one by indirect ELISA method. Here the expression level is higher in group2 as compared to control. Data presented are representative of three independent experiments performed in triplicates and expressed as Mean±S.D. ** and *** differs significantly at *p* <0.005 and <0.0005.
(TIF)

S3 Fig Expression level of Wnt and Hh pathway molecules in progression stage animals. (A) Expression level of Wnt and Hh pathway molecules in tissue lysates of control and DEN treated animals by ELISA method. (B) Expression level of Wnt and Hh pathway molecules in mRNA by RT-PCR method. (C) Expression level of MMP9 in the blood serum of control and DEN treated animals of different stages. Data presented are representative of three independent experiments performed in triplicates and expressed as Mean±S.D. *, ** and *** differs significantly at *p <0*.*05*, <0.005 and <0.0005.
(TIF)

S4 Fig Wound healing assay in the presence of purmorphamine (Pur). Hep3B cells were plated and treated with Pur. Before plating the cells, two parallel lines were drawn at the underside of the wells, to serve as marks demarcating the wound areas to be analyzed. Prior to inflicting the wound, the cells were 80–90% confluent. The media was aspirated off and replaced by the complete media with or without Pur. The wounds were observed using bright- field microscopy at various time points 0hr, 12hrs, 24hrs and multiple images of that areas were taken.
(TIF)

S5 Fig Expression levels of β-catenin post LiCl treatment. Animals were intraperitonially injected with LiCl(100mg/kg body weight) for 10 days after DEN+CCL4 treatment. Routine histopathology was performed on liver sections obtained from control and post LiCl treated animals. IHC staining of β-catenin was performed in treated liver tissue sections with control one. The corresponding IHC staining intensity graph is shown in the side panel of figures. Y-axis represents labeling index (%) visual score in each intensity graph.
(TIF)

S6 Fig Arrive checklist-Word version. (DOCX)

## Abbreviations

HCC: Hepatocellular carcinoma
TCGA: The Cancer Genome Atlas
WHO: World Health Organisation
EDTA: Ethylene Diaminetetra acetic acid
PCNA: Proliferative Cell Nuclear Antigen
sFRP1: secreted Frizzled Receptor Protein1
GSK3β: Glycogen Synthase Kinase 3β
ABTS: 2,2'-azino-bis(3-ethylbenzothiazoline-6-sulphonic acid).

## References

[1] Bosch FX, Ribes J, Díaz M, Cléries R. Primary liver cancer: worldwide incidence and trends. Gastroenterology 2004; 127: S5–S16 []. 15508102 10.1053/j.gastro.2004.09.011

[2] Friemel J, Rechsteiner M, Frick L, B€ohm F, Struckmann K, Egger M, et al Intratumor Heterogeneity in Hepatocellular Carcinoma; Clinical Cancer Res. 2015 4 15;21(8):1951–61. 10.1158/1078-0432.CCR-14-0122 25248380 10.1158/1078-0432.CCR-14-0122

[3] Anfuso B, El-Khobar KE, Sukowati CH, Tiribelli C. The multiple origin of cancer stem cells in hepatocellular carcinoma. Clinical Cancer Res Hepatol Gastroenterol. 2015 9;39 Suppl 1:S92–7. 10.1016/j.clinre.2015.05.011 Epub 2015 Jul 15. 26186879 10.1016/j.clinre.2015.05.011

[4] Hectors SJ, Wagner M, Bane O, Besa C, Lewis S, Remark R, et al Quantification of hepatocellular carcinoma heterogeneity with multiparametric magnetic resonance image; Scientific Reports 2017,5 26;7(1):2452 10.1038/s41598-017-02706-z 28550313 10.1038/s41598-017-02706-z PMC5446396

[5] Derwinger K, Kodeda K, Bexe-Lindskog E, Taflin H. Tumour differentiation grade is associated with TNM staging and the risk of node metastasis in colorectal cancer; Acta Oncologica, 2010, 49:1, 57–62, 10.3109/02841860903334411 20001500 10.3109/02841860903334411

[6] Keles I, Ceylan C, Aglamis E, Saglam HS, Karalar M, Coban S, et al Association between Tumor Stage and Grade and Mean Platelet Volume in Patients with Renal Cell Carcinoma. Clinical Medicine Research. Vol. 3, No. 2, 2014, pp. 36–39. doi: 10.11648/j.cmr.20140302.16

[7] Tabibi A, Parvin M, Abdi H, Bashtar R, Zamani N, Abadpour B. et al Correlation Between Size of Renal Cell Carcinoma and Its Grade, Stage, and Histological Subtype. Urol J (Tehran), 2007 Winter; 4(1):10–3. PMID: 17514606. 17514604

[8] Aaltonen LA, Hamilton SR. World Health Organization, International Agency for Research on Cancer. Pathology and genetics of tumours of the digestive system. Lyon Oxford: IARC Press; Oxford University Press (distributor); 2000. 314 p. p.

[9] Ingham P.W. and McMahon A.P. Hedgehog signaling in animal development: paradigms and principles (2001).Genes Dev. 15,3059–3087. 10.1101/gad.938601 11731473 10.1101/gad.938601

[10] Cadigan K. and Nusse R. Wnt signaling: a common theme in animal development (1997). Genes Dev. 11, 3286–3305. 9407023 10.1101/gad.11.24.3286

[11] Bowen KN, Thompson M D, Singh S, William C. Bowen W C Jr., Dar M J, et al Accelerated liver regeneration and hepatocarcinogenesis in mice overexpressing serine-45 mutant beta-catenin. Hepatology J 2010, 5 51(5): 1603–1613; 10.1002/hep.23538 10.1002/hep.23538 PMC2908905 20432254

[12] Omenetti A, Choi S, Michelotti G, Diehl A M: Hedgehog signaling in the liver; J Heptol, 2011; 10.1016/j.jhep.2010.10.003 21093090 10.1016/j.jhep.2010.10.003 PMC3053023

[13] Monga P S: Role of Wnt/βcatenin signaling in liver metabolism and cancer; Int J Biochem Cell Biol, 2011; 10.1016/j.biocel.2009.09.001 19747566 10.1016/j.biocel.2009.09.001 PMC2891959

[14] Pez F, Lopez A, Kim M, Wands JR, de Fromentel CC, Merle P. Wnt signaling and hepatocarcinogenesis: Molecular targets for the development of innovative anticancer drugs. J Hepatol. Volume 59, Issue 5, 11 2013, Pages 1107–1117. 10.1016/j.jhep.2013.07.001 23835194 10.1016/j.jhep.2013.07.001

[15] Sicklick JK, Li YX, Jayaraman A, Kannangai R, Qi Y, Vivekanandan P, et al Dysregulation of the Hedgehog pathway in human hepatocarcinogenesis. Carcinogenesis. 2006 4;27(4):748–57.Epub2005 Dec8. 10.1093/carcin/bgi292 16339184 10.1093/carcin/bgi292

[16] Huang S, He J, Zhang X, Bian Y, Yang L, Xie G et al; Activation of the hedgehog pathway in human hepatocellular carcinomas. Carcinogenesis vol.27 no.7 pp.1334–1340, 2006 10.1093/carcin/bgi378 16501253 10.1093/carcin/bgi378

[17] Heindryckx F, Colle I, Vlierberghe HV. Experimental mouse models for hepatocellular carcinoma research. Int J Exp Pathol. 2009 8; 90(4): 367–386. 10.1111/j.1365-2613.2009.00656.x 19659896 10.1111/j.1365-2613.2009.00656.x PMC2741148

[18] Newell P, Villanueva A, Friedman SL, Koike K, Llovet JM. Experimental models of hepatocellular carcinoma. J Hepatol. 2008 5; 48(5): 858–879. 10.1016/j.jhep.2008.01.008 18314222 10.1016/j.jhep.2008.01.008 PMC2990959

[19] Mansour MA, Bekheet SA, Al-Rejaie SS, Al-Shabanah OA, Al-Howiriny TA, Al-Rikabi AC, et al Ginger ingredients inhibit the development of diethylnitrosoamine induced pre malignant phenotype in rat chemical hepatocarcinogenesis model. Biofactors 2010; 36: 483–490. 10.1002/biof.122 20872761 10.1002/biof.122

[20] Abdo W, Hirata A, Shukry M, Kamal T, Abdel-Sattar E, Mahrous E, et al Calligonum comosum extract inhibits diethylnitrosamine-induced hepatocarcinogenesis in rats. Oncol Lett. 2015 8; 10(2): 716–722. 10.3892/ol.2015.3313 26622559 10.3892/ol.2015.3313 PMC4509362

[21] Martins-Filho SN, Paiva C, Azevedo RS and Alves VAF. Histological Grading of Hepatocellular Carcinoma—A Systematic Review of Literature. Front. Med. 2017 4:193 10.3389/fmed.2017.00193 29209611 10.3389/fmed.2017.00193 PMC5701623

[22] Suzuki T, Yano H, Nakashima Y, Nakashima O, Kojiro M. β-Catenin expression in hepatocellular carcinoma: A possible participation of β-catenin in the dedifferentiation process. Journal of Gastroenterology and Hepatology. 2002 17, 994–1000. 12167121 10.1046/j.1440-1746.2002.02774.x

[23] He J, Sheng T, Stelter AA, Li C, Zhang X, Sinha M, Luxon BA, et al Suppressing Wnt Signaling by the Hedgehog Pathway through sFRP-1. Journal of Biological Chemistry 2006 VOL. 281, NO. 47, pp. 35598–35602. 10.1074/jbc.C600200200 17035233 10.1074/jbc.C600200200

[24] Barandon L, Dufourcq P, Costet P, Moreau C, Allie`res C, Daret D, et al Involvement of FrzA/sFRP-1 and the Wnt/Frizzled Pathway in Ischemic Preconditioning. Circ Res. 2005;96:1299–1306. 10.1161/01.RES.0000171895.06914.2c 15920021 10.1161/01.RES.0000171895.06914.2c

[25] Ezan J, Leroux L, Barandon L, Dufourcq P, Jaspard B, Moreau C, et al FrzA/sFRP-1, a secreted antagonist of the Wnt-Frizzled pathway, controls vascular cell prolifration in vitro and *in vivo*. Cardiovasc Res. 2004;63:731–738. 10.1016/j.cardiores.2004.05.006 15306229 10.1016/j.cardiores.2004.05.006

[26] Dufourcq P, Couffinhal T, Ezan J, Barandon L, Moreau M, Daret D, et al FrzA, a secreted Frizzled Related Protein, induced angiogenic response. Circulation. 2002;106:3097–3103. 12473558 10.1161/01.cir.0000039342.85015.5c

[27] Arii S, Mise M, Harada T, Furutani M, Ishigami S, Niwano M, et al Overexpression of matrix metalloproteinase 9 gene in hepatocellular carcinoma with invasive potential. Hepatology 1996;24:316–322. 10.1053/jhep.1996.v24.pm0008690399 8690399 10.1053/jhep.1996.v24.pm0008690399

[28] Asai J, Takenaka H, Kusano KF, Ii M, Luedemann C, Curry C, et al Topical sonic hedgehog gene therapy accelerates wound healing in diabetes by enhancing endothelial progenitor cell-mediated microvascular remodeling. Circulation 2006 113:2413–24. 10.1161/CIRCULATIONAHA.105.603167 16702471 10.1161/CIRCULATIONAHA.105.603167

[29] Gering M1, Patient R. Hedgehog signaling is required for adult blood stem cell formation in zebrafish embryos. Dev Cell. 2005 3;8(3):389–400. 10.1016/j.devcel.2005.01.010 15737934 10.1016/j.devcel.2005.01.010

[30] Feldmann G1, Dhara S, Fendrich V, Bedja D, Beaty R, Mullendore M, et al Blockade of hedgehog signaling inhibits pancreatic cancer invasion and metastases: a new paradigm for combination therapy in solid cancers. Cancer Res. 2007 3 1;67(5):2187–96. 10.1158/0008-5472.CAN-06-3281 17332349 10.1158/0008-5472.CAN-06-3281 PMC3073370

[31] Suzuki H, Masuda N, Shimura T, Araki K, Kobayashi T, Tsutsumi S, et al Nuclear β-Catenin Expression at the Invasive Front and in the Vessels Predicts Liver Metastasis in Colorectal Carcinoma; Anticancer research 28: 1821–1830 (2008). 18630466

[32] Qu Y, Ray PS, Li J, Cai Q, Bagaria SP, Moren C, et al High Levels of secreted frizzled-related protein 1 correlate with poor prognosis and promote tumourigenesis in gastric cancer. European Journal of Cancer (2013) 49, 3718–3728. 10.1016/j.ejca.2013.07.011 23927957 10.1016/j.ejca.2013.07.011

[33] Saini S, Liu J, Yamamura S, Majid S, Kawakami K, Hirata H, et al Functional Significance of Secreted Frizzled-Related Protein 1 in Metastatic Renal Cell Carcinomas. Cancer Res 2009;69(17):6815–22. 10.1158/0008-5472.CAN-09-1254 19723665 10.1158/0008-5472.CAN-09-1254

[34] Herman JG, Baylin SB. Gene silencing in cancer in association with promoter hypermethylation. N Engl J Med 2003; 349: 2042–54. 10.1056/NEJMra023075 14627790 10.1056/NEJMra023075

[35] Jones PA, Baylin SB. The fundamental role of epigenetic events in cancer. Nat Rev Genet 2002; 3: 415–28. 10.1038/nrg816 12042769 10.1038/nrg816

[36] Ban KC, Singh H, Krishnan R, Seow HF. Comparison of the Expression of b-Catenin in Hepatocellular Carcinoma in Areas With High and Low Levels of Exposure to Aflatoxin B1. J. Surg. Oncol. 2004;86:157–163. 10.1002/jso.20051 15170655 10.1002/jso.20051

[37] Cheng WT, Xu K, Tian DY, Zhang ZG, Liu LJ, Chen Y. Role of Hedgehog signaling pathway in proliferation and invasiveness of hepatocellular carcinoma cells. Int. J Oncology. 34: 829–836, 2009. 10.3892/ijo_00000209 19212688

[38] Sun X, Liu Y. Activation of the Wnt/β-catenin signaling pathway may contribute to cervical cancer pathogenesis via upregulation of Twist. Oncol Lett. 2017 10;14(4):4841–4844. 10.3892/ol.2017.6754 Epub 2017 Aug 14. 29085489 10.3892/ol.2017.6754 PMC5649628

[39] Kneidinger N, Yildirim AÖ, Callegari J, Takenaka S, Stein MM, Dumitrascu R, et al Activation of the WNT/β-catenin pathway attenuates experimental emphysema. Am J Respir Crit Care Med. 2011 3 15;183(6):723–33. 10.1164/rccm.200910-1560OC Epub 2010 Oct 1. 20889911 10.1164/rccm.200910-1560OC

[40] Quan H, Zhou F, Nie D, Chen Q, Cai X, Shan X, et al Hepatitis C virus core protein epigenetically silences SFRP1 and enhances HCC aggressiveness by inducing epithelial-mesenchymal transition. Oncogene 33(22): 2826–2835, 2014 10.1038/onc.2013.225 23770846 10.1038/onc.2013.225

[41] Kaur P, Mani S, Cros MP, Scoazec JY, Chemin I, Hainaut P, et al Epigenetic silencing of sFRP1 activates the canonical Wnt pathwayand contributes to increased cell growth and proliferation in hepatocellular carcinoma; Tumor Biology, 2012, Volume33, Issue-2, pp 325–336. 10.1007/s13277-012-0331-5 22351518 10.1007/s13277-012-0331-5

[42] Shih YL, Shyu RY, Hsieh CB, Lai HC, Liu KY, Chu TY, et al Promoter methylation of the secreted frizzled-related protein 1 gene sFRP1 is frequent in hepatocellular carcinoma; Cancer, ACS, 2006;107:579–90. 10.1002/cncr.22023 16795071

[43] Davaadorj M, Imura S, Satio Y, Morine Y, Ikemoto T, Yamada S, et al Loss of sFRP1 expression is associated with poor prognosis in hepatocellular carcinoma; Anticancer research, 2016 vol.36 no. 2 659–664. 26851021

[44] Kim TH, Chang JS, Park KS, Park J, Kim N, Lee JI, et al Effects of exercise training on circulating levels of Dickkpof-1 and secreted frizzled- related protein-1 in breast cancer survivors: A pilot single- blind randomized controlled trial. Plos One,2017, 10.1371/journal.pone.0171771. PMC5298304 28178355

[45] Jonas S, Bechstein WO, Steinmuller T, Herrmann M, Radke C, Berg T, et al Vascular invasion and histopathologic grading determine outcome after liver transplantation for hepatocellular carcinoma in cirrhosis. Hepatology. 2001;33:1080–1086. 10.1053/jhep.2001.23561 11343235 10.1053/jhep.2001.23561

