# Supplementary figures and images for: The molecular connection of histopathological heterogeneity in hepatocellular carcinoma: A role of Wnt and Hedgehog signaling pathways
Source: PLoS One. 2018 Dec 4;13(12):e0208194. doi: 10.1371/journal.pone.0208194 (PMC6279049; doi:10.1371/journal.pone.0208194)

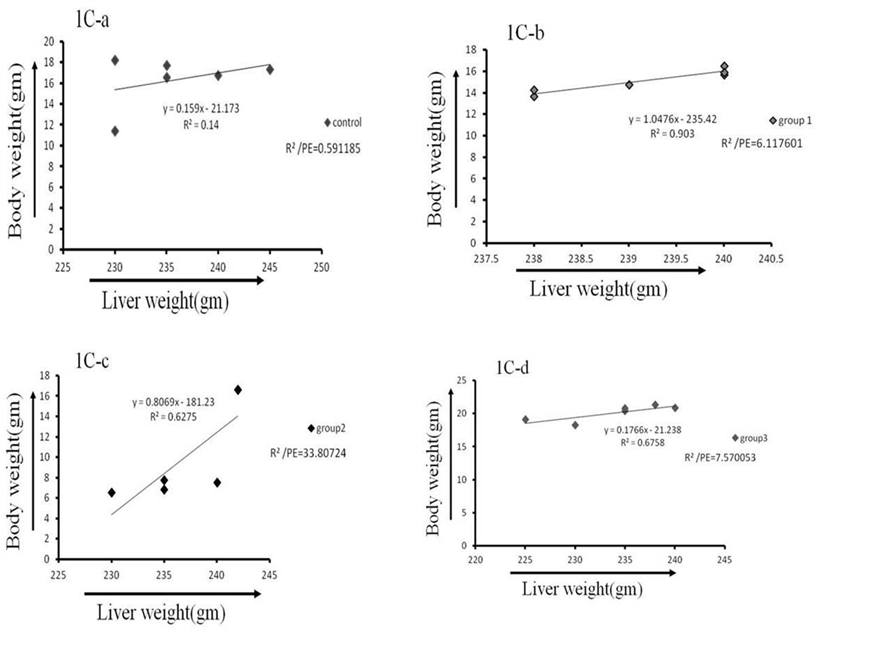

Supplement: S1 Fig — The graphs revealed that there is a significant correlation (R2/P.E>6) between liver weight and body weight in group1, 2, and 3 animals. To determine extent of correlation, probable error (PE) of coefficient of correlation was calculated with the formula PE = 0.6745(1-R2)/(6)1/2 (Fig 1C(a-d)). Panel (a) shows linear regression analysis graph for control, panel (b) for group1, panel (c) for group2, and panel (d) for group3 animals. These results depict considerable regeneration of liver taking place after DEN and CCl4 treatment. (TIF) [file pone.0208194.s001.tif]

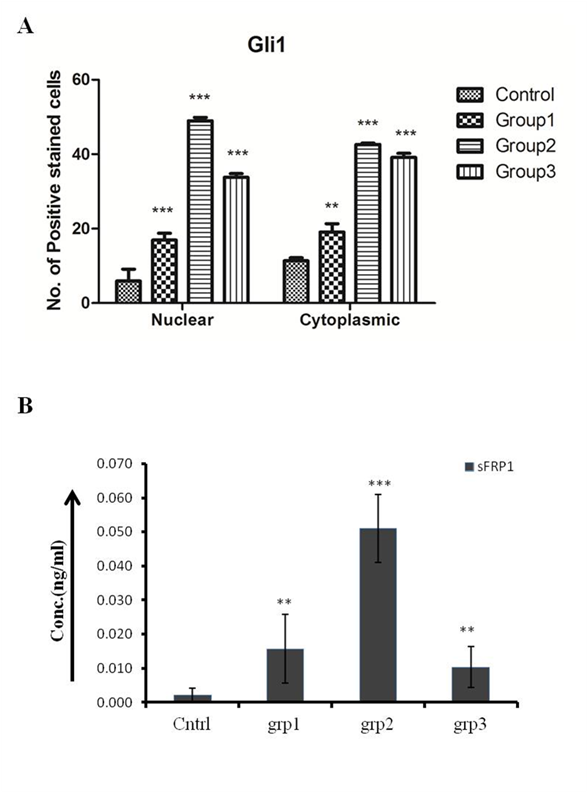

Supplement: S2 Fig — (A) Graph showing the number of positive stained cells for nuclear, and cytoplasmic staining of Gli1. The number of positive cells were counted belonging to five different fields of five different sections of control, group1, group2 and group3 animals. (B) Expression level of sFRP1 in the rat tissue lysates of different groups (group1,2 & 3) with control one by indirect ELISA method. Here the expression level is higher in group2 as compared to control. Data presented are representative of three independent experiments performed in triplicates and expressed as Mean±S.D. ** and *** differs significantly at p <0.005 and <0.0005. (TIF) [file pone.0208194.s002.tif]

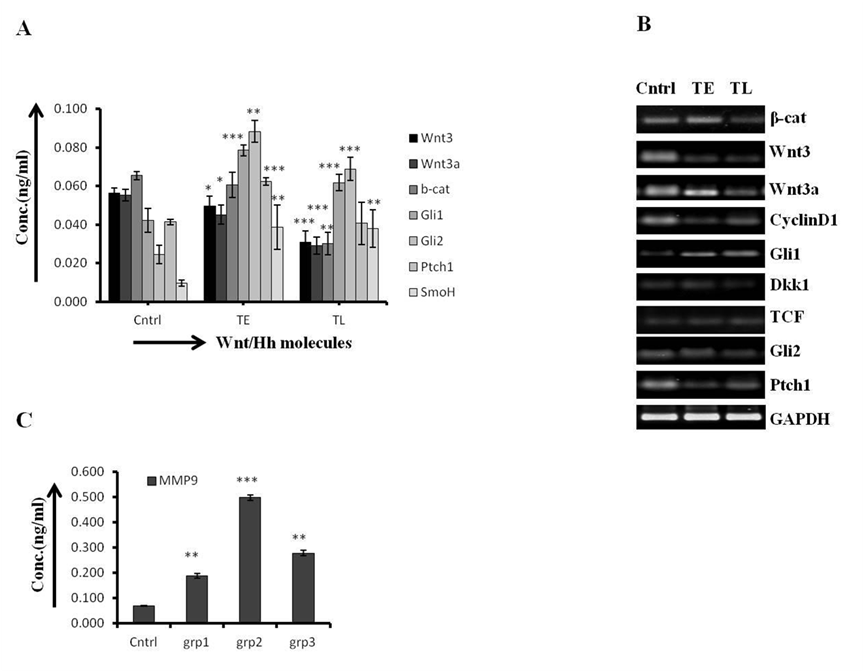

Supplement: S3 Fig — (A) Expression level of Wnt and Hh pathway molecules in tissue lysates of control and DEN treated animals by ELISA method. (B) Expression level of Wnt and Hh pathway molecules in mRNA by RT-PCR method. (C) Expression level of MMP9 in the blood serum of control and DEN treated animals of different stages. Data presented are representative of three independent experiments performed in triplicates and expressed as Mean±S.D. *, ** and *** differs significantly at p <0.05, <0.005 and <0.0005. (TIF) [file pone.0208194.s003.tif]

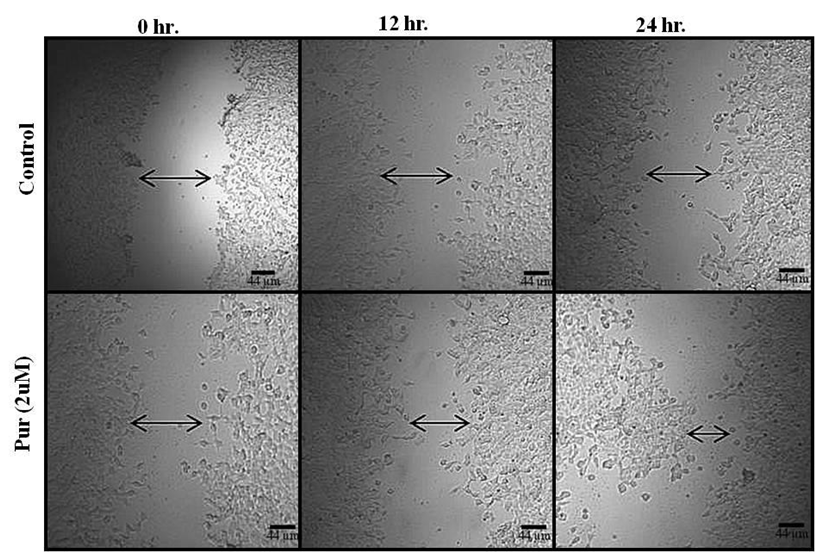

Supplement: S4 Fig — Hep3B cells were plated and treated with Pur. Before plating the cells, two parallel lines were drawn at the underside of the wells, to serve as marks demarcating the wound areas to be analyzed. Prior to inflicting the wound, the cells were 80–90% confluent. The media was aspirated off and replaced by the complete media with or without Pur. The wounds were observed using bright- field microscopy at various time points 0hr, 12hrs, 24hrs and multiple images of that areas were taken. (TIF) [file pone.0208194.s004.tif]

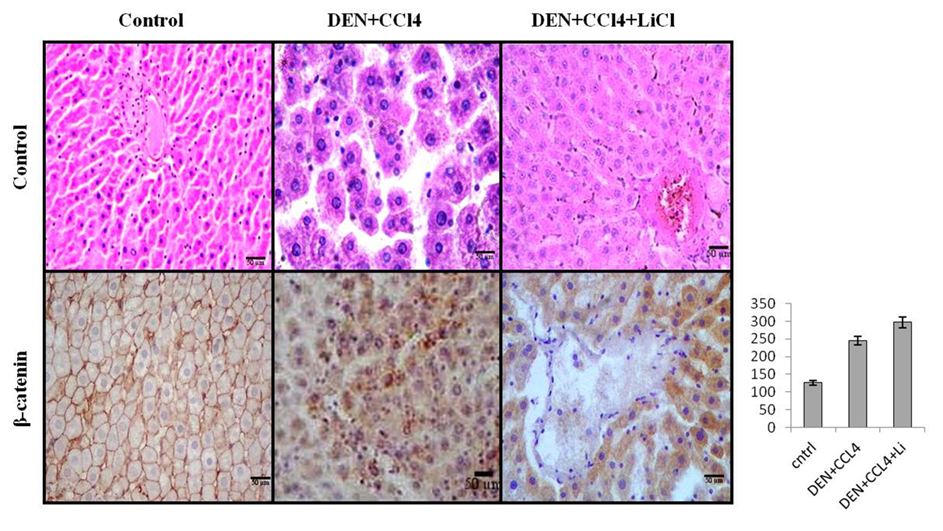

Supplement: S5 Fig — Animals were intraperitonially injected with LiCl(100mg/kg body weight) for 10 days after DEN+CCL4 treatment. Routine histopathology was performed on liver sections obtained from control and post LiCl treated animals. IHC staining of β-catenin was performed in treated liver tissue sections with control one. The corresponding IHC staining intensity graph is shown in the side panel of figures. Y-axis represents labeling index (%) visual score in each intensity graph. (TIF) [file pone.0208194.s005.tif]
