# Supplementary material for: The molecular connection of histopathological heterogeneity in hepatocellular carcinoma: A role of Wnt and Hedgehog signaling pathways
Source: PLoS One. 2018 Dec 4;13(12):e0208194. doi: 10.1371/journal.pone.0208194 (PMC6279049; doi:10.1371/journal.pone.0208194)
Supplement: S6 Fig — (DOCX) [file pone.0208194.s006.docx]

**The ARRIVE Guideline Checklist**

1. **Title**: The molecular connection of histopathological heterogeneity in hepatocellular carcinoma: a role of Wnt and Hedgehog signaling pathways
2. **Abstract:**

**Backgraound**:Hepatocellular carcinoma (HCC) is leading cause of cancer-related mortality and is categorized among the most common malignancies around the world. It is a heterogeneous tumor, which shows significant degree of histopathological heterogeneity. Despite the apparent histopathological diversity there has been very little distinct correlation between histopathological features and molecular aberrations, particularly when the role of Wnt and Hedgehog signaling pathways are considered. The role of Wnt and Hh pathways in relation to HCC behavior viz. histopathological heterogeneity and aggressiveness is not known. Determining the sequential molecular changes and associated histopathological characteristic during HCC initiation, promotion, and progression would probably lead to a better treatment and prognosis. **Methods:** N-Nitrosodiethylamine (DEN) induced HCC model in male Wistar rats were established to study the expression level of Wnt and Hh pathway molecules during different stages of hepatocarcinogenesis. Their expression levels were checked at mRNA and protein levels at initiation, promotion, and progression stages of HCC. Principal findings: In the present study we identified the comprehensive change in the expression pattern of Wnt and Hh pathway molecules in DEN induced rodent hepatocarcinogenesis model. **Results:** Our results demonstrate that β-catenin/CTNNB1 plays important role in tumor initiation and promotion by stimulating tumor cell proliferation and the activated Wnt signaling in early stage of HCC is associated with well-differentiated histological pattern. The Hh activity is increased during the early promotion stage of hepatocarcinogenesis. The increased activity of both Wnt & Hh pathways during promotion stage is associated with moderately-differentiated histological pattern and was simultaneously linked with an increased expression of MMP9. Furthermore, our data demonstrated that during the progression stage Wnt pathway is modestly down-regulated but the Hh pathway activity sustained which in turn is associated with aggressive and invasive phenotype and poorly-differentiated histopatholog. **Conclusion:** Our data uncovers the grade related expression of Wnt and Hh pathway molecules and the potential utility of these molecular signatures in daily clinical practice is to decide best therapy according to patients characteristic. Additionally, our data offer insight into the interaction between Wnt and Hh pathways which triggers HCC development and progression.

1. **INTRODUCTION:**

**Background**

a. Hepatocellular carcinoma (HCC) is leading cause of cancer-related mortality and is categorized among the most common malignancies around the world[1]. HCC is a morphologically and clinically heterogeneous tumor and it also shows a significant degree of histopathological heterogeneity[2-4]. This heterogeneous nature of HCC pose serious problem for its therapeutic management. Particularly, there is obvious and frequent histopathological changes observed at different stages of HCC. /ß-catenin and Hedgehog (Hh) signaling pathways are two fundamental developmental pathways which are found to be involved in regeneration of liver after injury. The role of these pathways has been indicated in several human cancers. Interestingly, there are reports which document the cross talk between these two pathways during development and carcinogenesis. Since the role of Wnt and Hh pathway interaction has not been studied in hepatocellular carcinoma (HCC), we decided to investigate if there is any functional interaction (positive or negative) between these two pathways in HCC. Motivation and context for the study: In order to study the role the two developmental signaling pathways at different stages of hepatocarcinogenesis we planned to do the DEN induced multistep hepatocarcinogenesis experiment. Experimental approach and rationale: We used Male Wistar rats for our experiment. As multistep hepatocarcinogenesis study can only be done in animal models. Additionaly, rat liver is a frequent target for the development of chemically induced cancer in rodents, and it is the most commonly used experimental model for investigating multistage carcinogenesis in vivo. Furthermore, sacrificing animals at different stages of carcinogenesis (Initiation, Promotion, and Progression) allowed us to study the role of Wnt and Hh signaling pathway molecules at these stages. So could be done in animal models only.

b. **The relevance of animal species and model used**: We preferred to use rat model over mouse, because: first, many mouse strains seem to yield high rates of spontaneous liver tumours. Spontaneous liver tumours in rats are rarer. Particularly use of male wistar rats as a frequent model for chemical induced hepatocarcinogenesis has been already established. Furthermore, in the rat liver carcinogenesis models, a variety of enzyme-altered condition has been studied for their relevance to preneoplastic and neoplastic developments. For example, GST-P has been utilized for the identification of liver preneoplastic focal lesions and could be detected by immunohistochemistry. Relevance of the study to human biology: One of the important diagnostic features for almost all malignancies including HCC is cytological properties which includes the cellularity, arrangement-pattern, and nucleo-cytoplasmic details. Based on these properties HCC can be categorized into well-differentiated, moderately-differentiated, poorly-differentiated, and undifferentiated lesions. The widespread molecular nature of HCC across different grades is not known. In the present study we identified the comprehensive change in the expression pattern of Wnt and Hh pathway molecules in DEN induced rodent hepatocarcinogenesis model. The differential expression of Wnt and Hh pathway molecules could discriminate the differences in the histopathological grades from pre-neoplastic lesion to advanced HCC. Simple microscopic examination is not enough to differentiate between different grades of HCC. A significant difference in the molecular signature of each histopathological grade during hepatocarinogenesis would open a window for grade specific treatment option. Additionally, molecular signatures associated with specific grades would in turn improve the histopathological grading system.

1. **Objective**: to check the expression level of Wnt and Hegdehog signaling pathways at different steps of DEN induced hepatocarcinogenesis model of male Wistar rats. Seconday objective: To corroborate our in vivo findings obtained with the data obtained through analysis of biospecimens of HCC patients belonging to different stages. Hypthesis: The Wnt and Hh signaling pathways are known to be involved in various steps of liver regeneration. Although Wnt and Hh are unrelated proteins, they have some fundamental similarities which suggest the probability of common evolutionary origin of these pathways. Since these pathways are involved in HCC, we hypothesize that interaction between these two pathways are crucial during hepatocarcinogenesis. By exploring these interactions we should be able to achieve significant advancement into understanding of the role of these pathways in HCC.

**METHODS:**

1. **Ethical Statement:** All the experimental protocols were approved by the Institutional Animal Ethics Committee (IAEC, KIIT School of Biotechnology,Bhubaneswar, India). Relevant licences: 1. Name and address of establishment: School of Biotechnology, KIIT University. Bhubaneswar-751024, Odisha. 2. Registration number and date of registration: 1577/PO/ac/11/CPCSEA Date: 09.05.2013.
2. **Study design:** a. n=6 b. The rats were randomly and evenly allocated into four groups, six rats in each group. All animals were acclimatized for two weeks before starting the experiment. c. Male wistar rats of nearly 6 weeks old (120 to 150 gm body weight) was used in this study. Animals were housed in the KSBT animal house facility with a 12:12 hr L: D cycle (lights on at 7.00 AM). Food and water were provided ad libitum. Experimental unit: 6 rats per group and were kept as 3 rats in one big cage (2 cage per group).
3. **Experimental details:** a. The two stage chemically induced hepatocarcinogenesis protocol was followed which included initiation and promotion stage. Initiation was done with a chemical carcinogen DEN and injected Intraperitoneally (IP) 100mg/kg body weight of the wistar rats followed by promotion with an another chemical CCL4 IP 2ml/kg body weight. b. DEN was injected once in a week upto 3 weeks and after one week of recovery period, CCl4 was injected twice in a week upto 19th weeks. c. Animals were injected in the laboratory sterile condition of the animal house in KSBT. d. Intraperitoneal (IP) injection is used for toxicology and other experiments where other routes are not considered suitable. Compounds absorbed IP will pass through hepatic circulation prior to distribution to other organs. Intraperitoneally applied drug forms are supposed to be located in the peritoneal cavity and aqueous systems will mix with the relatively small amount of peritoneal fluid. 100mg/kg body weight of DEN is known to cause hepatocarcinogenesis in rodents when injected for three consecutive weeks.
4. **Experimental animals detail:** a. Animal used: Male Wistar rats Development stage: 6 week age; body weight 120 to 150 gm. b. Source of animals: Name, address and registration number of breeder from whom animals were acquired: Charles River Laboratories International, Inc. 251 Ballardvale Street. Wilmington, MA, USA 01887 International strain nomenclature:Wistar Rat Nomenclature: Crl:WI Strain Code: 003 Origin: To Scientific Products Farm, Ltd. [predecessor of Charles River United Kingdom] in 1947 from Wistar Institute. To Charles River North America in 1975 from Charles River UK. Coat Color: White (Albino).
5. **Animal housing and husbandary:** a. Animals were housed in the KSBT Animal House Facility, Lab no 106. b. Animals were housed in standard room temperature/ humidity conditions and environment (12hr light/dark cycle). All animals were provided standard pellet diet and water ad libitum.
6. **Sample size:** a. n=6 rats in each group total n=24 rats b. The animals were arrived from National Centre for Laboratory Animal Sciences, Hyderabad, India. c. None
7. **Animal allocation:** a. Rats were randomly and evenly allocated into four groups, six rats in each group. All animals were acclimatized for two weeks before starting the experiment. b. At first Group1,2,3 animals were treated with DEN once in a week upto 3 weeks. After recovery of one week, they treated with CCl4 twice in a week upto 19^th^ weeks . Control rats were untreated.
8. **Primary experimental outcomes:** Biochemical assay in blood serum of animals to check liver functional status. Markers for hepatocarcinogenesis like GSTP1 expression level and PCNA expression level. Immunohistochemical analysis of various Wnt and Hh pathway molecules. Secondary experimental outcomes: Wnt and Hh signaling pathways funtional status and corresponding histopathological changes at different steps of DEN induced hepatocarcinogenesis.
9. **Statistical methods used:** a. The data presented was the Mean±SD of three independent experiments. Changes in gene expression and cytoloplasmic nuclear staining were analyzed by two way analysis of varience (ANOVA) using Graph-pad Prism5. The fold change of m-RNA was used as variables to copare samples between different treatment groups. Statistical analysis for ELISA readouts of tissue lysates were carried out using two tailed analysis. b. **p* <0.05, ***p* <0.005, ****p* <0.0005 were considered to be significant. C. N/A

**RESULT:**

1. **Baseline data:** Health status of animals:

- Male Wistar rats of nearly 6weeks old
- 120-150g body weight,
- healthy in physical condition.

After treatment the body weight of each rat was increased as liver weight was increased but the food and water intake was gradually decreased and it was started from the 2^nd^ dose of DEN. Rats were became week , all rats were alive during the whole experiment period.

1. **Numbers analysed:** All the animals belonging to the four different groups were analysed and the data represented average of all animals in each group. a. Control: n=6; Group1:n=6; Group2:n=6; Group3:n=6. b. All 24 animals were analysed in our experimentsand out of 24, 18 animals were used for DEN+CCl4 treatment under different stages of hepatocarcinogenesis (Initiation, Promotion, Progression Stages). The tumor incidence rate was 100% as all 18 animals showed tumor in their liver.

1. **Outcomes and estimation:** From all 18 DEN+CCl4 treated rats we collected blood serum then we estimated the bilirubin assay by billirubin assay kit (SIGMA-ALDRICH, MAK126).The bilirubin concentration was calculated with the formula[ {(A530) sample-(A530)blank}/{(A530)calibrator-(A530)water}]*(5mg/dL) where: (A530)sample= value of the sample(Total), (A530)blank=value of the sample blank, (A530)calibrator=value of the calibrator, (A530)water= value of the water control, 5mg/dL= equivalent bilirubin concentration of the calibrator when assay performed as indicated. The linear regression and extent of correlation between body weight and liver weight of animals of control, group1, group2, and group3 , probable error (PE) of coefficient of correlation were calculated with the formula PE= 0.6745(1-R^2^)/(6)1/2. The intensity score of each IHC images was calculated by Immuno-Ratio Analysis software.
2. **Adverse events:** well established experimental protocols were followed for DEN+CCl4 induced hepatocarcinogenesis in male wistar rats. a. none b. None.

**DISCUSSION**

1. **Interpretation/scientific implications:** a. HCC is a histopathologically heterogeneous disease and its heterogeneous nature poses serious problem for its therapeutic management. The range of cellular differentiation of HCC extent from well differentiated to poorly differentiated types. Despite the apparent histopathological diversity they has been very little distinct correlation between histopathological features and molecular aberrations. In our study we identified the comprehensive change in the expression pattern of Wnt and Hh pathway molecules in DEN induced rodent hepatocarcinogenesis model. As simple microscopic examination is not enough to differentiate between different grades of HCC. A significant difference in the molecular signature of each histopathological grade during hepatocarcinogenesis would open a window for grade specific treatment option. Additionally molecular signatures associated with specific grades would intern improve the histopathological grading system. Conclusively, our data provide a foundation for grade specific treatment options by therapeutically targeting Wnt/Hh pathways. b. None. c. We have used statistically significant no. of animals to fulfill our objectives. As our experiment was stage specific so it can only be done in animal models. We already used lesser no. of animals depecting that animal experiment can be performed in minimum no. of statistically significant animals if the established experimental protocol is followed carefully.
2. **Generalisability/translation:** Our data uncover the grade-related expression pattern of Wnt and Hh pathway molecules which is simultaneously associated with a shift in histological pattern from well-differentiated to poorly-differentiated. Our data is also corroborated by human biospecimens data available at TCGA database and HCC patient samples belonging to different stages of disease.
3. **Funding:** Department of Biotechnology, Government of India (http://www.dbtindia.nic.in/) , Grant No.- BT/Bio-CARe/07/567/2011-12 provided financial support to carry out this work.
